# REACH: a reproducible benchmark for inter-lineage rare malignant cell detection in single-cell RNA sequencing

**DOI:** 10.3389/fbinf.2026.1878538

**Published:** 2026-10-19

**Authors:** Moram Venkata Satya Jaswanth, Amruthaluri Gavin, Padarthi Aakash, Muttha Tejesh Kumar, Abhijit Dasgupta

**Affiliations:** 1 Department of Computer Science and Engineering, School of Engineering and Sciences, SRM University-AP, Amaravati, Andhra Pradesh, India; 2 Artificial Intelligence Technology Institute, SRM University-AP, Amaravati, Andhra Pradesh, India

**Keywords:** imbalanced ranking, rare malignant cell detection, reproducible benchmarking, single-cell RNA sequencing, tumor heterogeneity

## Abstract

Rare malignant cells in single-cell RNA sequencing (scRNA-seq), including circulating tumor cells (CTCs), minimal residual disease (MRD) cells, and small malignant populations in heterogeneous tumor microenvironments (TME), represent an important clinical challenge. These cells occur at very low frequency, requiring separation from a large non-malignant background. Existing methods show inconsistent evaluation, using different datasets, label definitions, prevalence settings, scoring strategies, and failure handling. We present Rare-cell Evaluation Across Cancer Heterogeneity (REACH), treating inter-lineage rare malignant cell detection as an imbalanced ranking task. REACH benchmarks CNV-supported malignant cells (P_HC) against immune, stromal, and normal epithelial cells (B_HC), modeling the CTC and MRD screening setting. REACH excludes intra-lineage state discovery, including drug-tolerant persisters, epithelial–mesenchymal transition (EMT), and transitional malignant states, evaluating these separately in Track F. REACH combines confidence tiered multi-arm labels with five evaluation tracks, covering controlled real spike-ins, synthetic Splatter stress tests, null background controls, natural blood/CTC prevalence, and supervised label noise diagnostics. Applying standardized method wrappers, fallback-aware metrics, non-parametric statistics, and audited figure regeneration, REACH provides a reproducible evaluation framework. REACH evaluates 10 computational methods on 10 curated scRNA-seq cancer datasets containing 359,739 cells. This benchmark produces 11,250 performance measurements across 1,125 benchmark tasks. The supervised highly variable gene logistic regression ceiling achieves a median average precision of 1.0, indicating strong expression differences between tumor and non-tumor cells. Current unsupervised methods recover only part of this signal. CaSee, serving as a cancer/normal cell scorer rather than a rare-cell detector, reaches a median average precision of 0.512 (0.606 excluding 20 degenerate units). FiRE, the strongest published inter-lineage detector, achieves a median average precision of 0.313. Statistical testing confirms significant performance differences among methods. REACH shows strong context dependence, with no method performing best across all tumor types. Track F further shows performance depending strongly on the inter-lineage malignant-versus-microenvironment contrast. Restricting the background to normal epithelial cells of the same lineage, nine of ten methods fall to near-chance average precision. These results indicate that inter-lineage leaderboards should not be extrapolated to intra-lineage rare-cell discovery.

## 1 Introduction

Tumor samples are often highly mixed. Along with dominant malignant and non-malignant populations, they may contain small groups of cells that are biologically important but difficult to observe in bulk measurements. Single-cell RNA sequencing (scRNA-seq) allows us to look at such populations of cells separately and to investigate tumor heterogeneity with an unprecedented level of detail (Papalexi and Satija, 2018; Tirosh and Suva, 2024). This is especially important when it comes to circulating tumor cells, metastatic intermediates, minimal residual disease cells, drug-tolerant persisters, and small malignant compartments in the tumor microenvironment. These populations can be low frequency, e.g., contribute to 1% or less in a given dataset (Torre et al., 2018; Gao et al., 2021). That makes them challenging to identify not only in biological terms but also in statistical ones. They can be more difficult to extract if rare cells are in a more diluted cluster. If these cells are rare, mixed with neighboring cells expressing related cell types or have low confidence from expression and copy number evidence, they become nearly impossible to identify (Märtens et al., 2023; Stegle et al., 2015; Ren et al., 2018).

This is particularly challenging for cancer datasets where the label malignant may not be clear cut. A cell can be classified as malignant based on copy number alteration, cancer-associated expression signatures, somatic mutation evidence, neighborhood structure, or expert annotations (Gao et al., 2021; Gas et al., 2022; Nofech-Mozes et al., 2023). However, in practice, the evidential sources may not be fully congruent. For some cells, there is strong evidence of expression but weak support by copy number, whereas others can be hard to classify if they are near non-malignant populations. This sets rare malignant cell detection apart from its cousin cell-type annotation, where the expected classes are usually more clearly delineated. Moreover, the problem is extremely imbalanced as there are frequently many more background cells surrounding the malignant cells of interest (Johnson and Khoshgoftaar, 2019; Altalhan et al., 2025).

This imbalance also raises questions about how methods should be assessed. The area under the receiver operating characteristic curve (AUROC) is widely used as a performance metric for machine learning classification and ranking tasks, but it becomes unwieldy and confusing if the positive class size shrinks very small. If true rare malignant cells are not ranked anywhere near the top of the list for a method, it may obtain a quite reasonable AUROC. Due to the high imbalance ratio, precision-recall-based measures are often more meaningful in this setting because they reflect whether rare positive cells are recovered among the highest-ranked predictions (Davis and Goadrich, 2006; Saito and Rehmsmeier, 2015). However, performance should never be evaluated on just a single statistic. Changing the label definition, data distribution, null behavior, method failure, and statistical error complicates the interpretation of benchmark results.

There have been multiple scRNA-seq benchmarks that have already been created to test batch effect correction, clustering, cell-type annotation, and rare-cell discovery. These benchmarks serve as useful reference standards for method comparison. Nevertheless, rare malignant cell detection requires a more tailored assessment plan. The targeted population may be very small; the label may require multiple types of biological evidence; and the same approach may not perform uniformly across tumor types, sequencing platforms, or prevalence settings (Tran et al., 2020; Wang et al., 2022; Tian et al., 2019; Abdelaal et al., 2019). Thus, a benchmark for this task should not return just one best method. It should use common rules to compare methods, separate supervised reference performance from unsupervised ranking performance, log failed or unstable runs, include null-control analysis, and report results in a cancer- and dataset-specific manner (Cao et al., 2021).

We herein present Rare-cell Evaluation Across Cancer Heterogeneity (REACH), a reproducible benchmark for inter-lineage rare malignant cell detection in scRNA-seq data (Figure 1). REACH formulates the detection of rare malignant cells as an inter-lineage imbalanced ranking problem: a method is expected to assign higher scores to high-confidence malignant cells [positive high confidence (P_HC)—copy number variation (CNV)-supported, tumor-intrinsic] than to high-confidence background cells [background high confidence (B_HC)—immune, stromal, and normal epithelial]. This malignant versus tumor microenvironment (TME) contrast models the circulating tumo -cell (CTC) and minimal residual disease (MRD) screening regime, whereas it is explicitly not a test of intralineage substate discovery.

**FIGURE 1:**
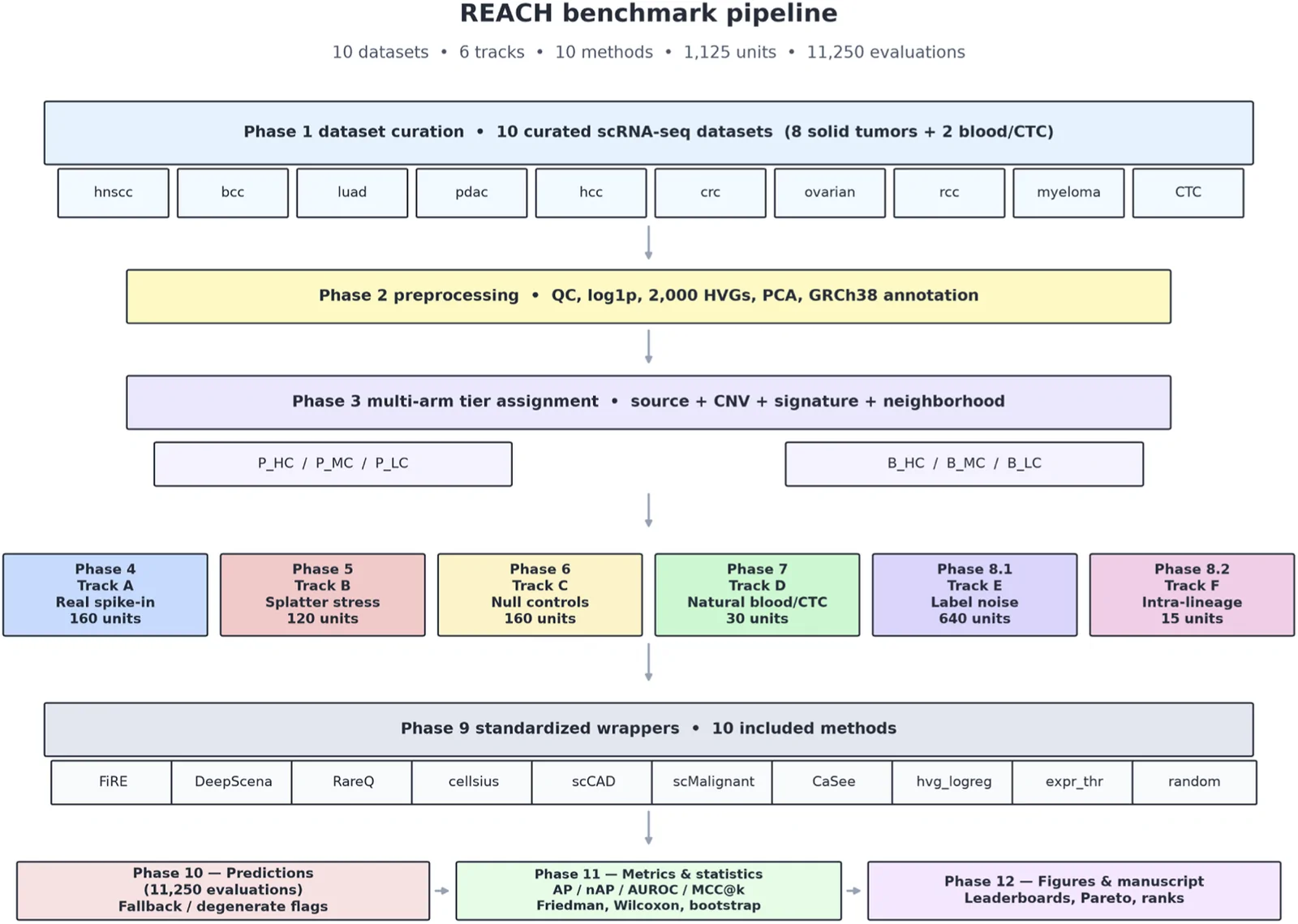
REACH benchmark pipeline. Ten curated scRNA-seq datasets are ingested; preprocessed; assigned confidence tiered malignant/background labels; transformed into six evaluation tracks (A–F); evaluated through standardized wrappers for ten methods; and summarized by metrics, statistical tests, and figures. Each method is evaluated on 1,125 benchmark units, yielding 11,250 method–unit evaluations across ten methods. The schematic mirrors the Phase 0–12 file contracts documented in docs/architecure.md of our GitHub repository.

Five primary evaluation tracks (A–E) analyze different aspects of method behavior on the inter-lineage malignant-versus-TME contrast (Figure 2): controlled real spike-ins (Track A, primary endpoint), synthetic Splatter stress (Track B, secondary), background-only null controls (Track C), natural blood/CTC prevalence (Track D), and supervised label noise diagnostics (Track E). A sixth intra-lineage diagnostic track (Track F) masks the TME contrast to test within-lineage detection (see Section 3.6). The current audited REACH snapshot includes 10 datasets, 10 methods, and 11,250 individual method performance measurements, and the framework is extensible to new datasets and methods.

**FIGURE 2:**
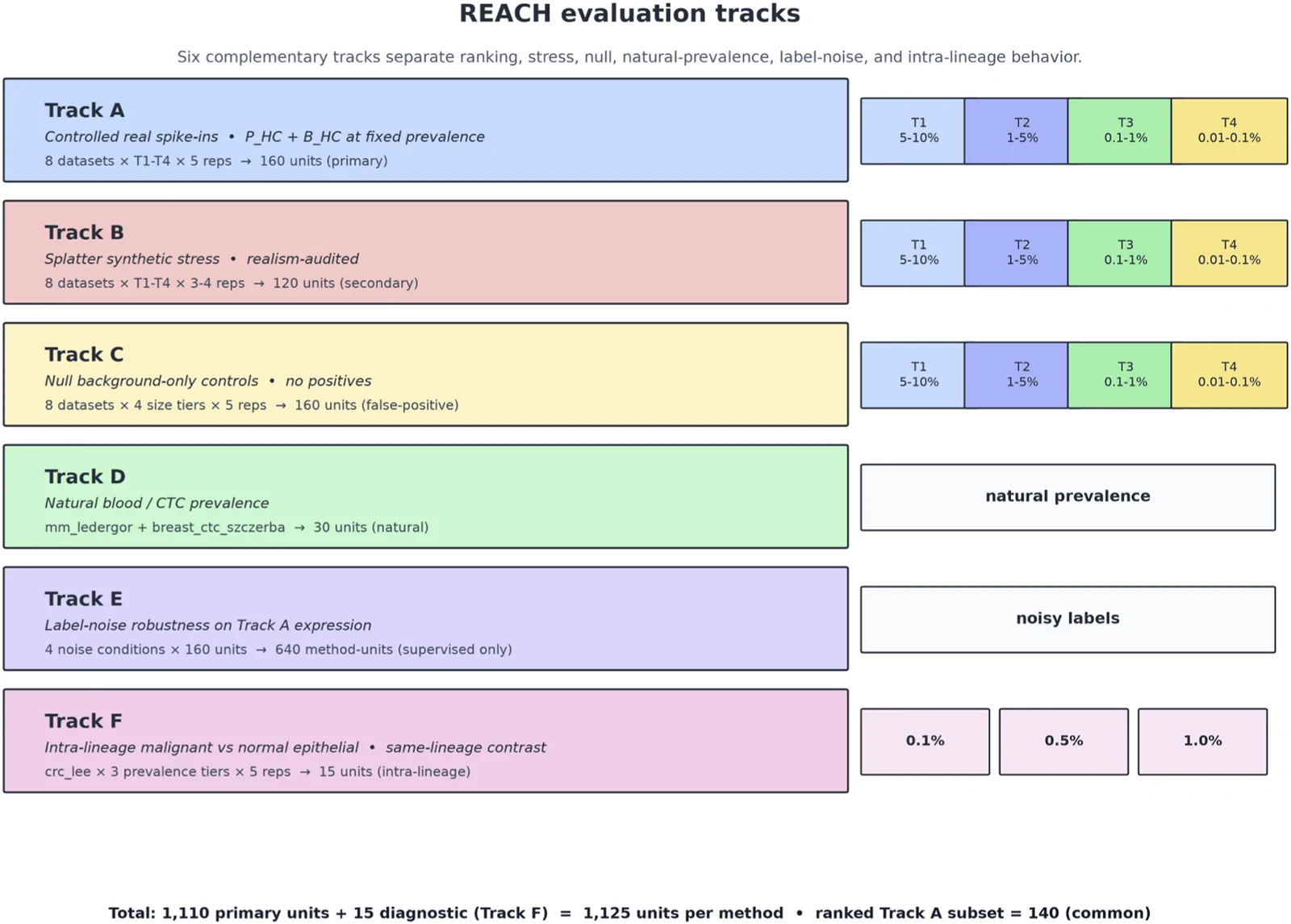
REACH evaluation tracks. Tracks A–E are built on the inter-lineage malignant-versus-TME contrast. Track F is the intra-lineage diagnostic in which the background is restricted to normal epithelial cells of the same tissue origin as the malignant positives, masking the global TME noise; it is constructed from crc_lee only and is not blended into the primary leaderboard. There are 1,125 units per method total across Tracks A–E, plus 15 Track F units.

The results show that many high-confidence malignant and background labels contain gene expression differences that can be recovered by a supervised model. However, current unsupervised and rarity-based methods recover only part of this separation on the primary Track A endpoint (Abdelaal et al., 2019; Mahalanabis et al., 2022). REACH also shows that method performance changes across datasets and evaluation tracks. A method that performs well in one tumor context may not remain the strongest method in another (Xu et al., 2024; Ye et al., 2025). These findings support the need for a reproducible benchmark that evaluates rare malignant cell detection in relation to dataset context, rarity level, failure behavior, and evaluation condition.

REACH benchmarks the interlineage problem of calling scarce CNV-supported malignant cells P_HC out of a heterogeneous non-malignant background B_HC: immune, stromal, and normal epithelial cells, modeling the CTC and MRD screening regime. It does not evaluate intra-lineage sub-state discovery (e.g., drug-tolerant persisters, EMT, and transitioning cells), which requires a same lineage contrast; the new Track F provides a first intra-lineage probe and confirms that inter-lineage leaderboards cannot be extrapolated to within-lineage detection.

## 2 Related work

### 2.1 Rare-cell discovery in scRNA-seq

Finder of Rare Entities (FiRE) assigns a density-derived rarity score to each cell and is included in REACH as one of the ranked published comparators (Märtens et al., 2023). The Cell Subtype Identification from Upregulated gene Sets (CellSIUS) method leverages upregulated gene sets to detect rare subpopulations in single-cell RNA sequencing data with high specificity (Wegmann et al., 2019). Single-molecule RNA fluorescence in situ hybridization shows that detecting rare expression depends on two factors: the studied gene set and the number of analyzed cells (Torre et al., 2018). Further analysis of cellular expression using deep clustering and anomaly detection scorers, such as Deep Single-Cell Ensemble Network Analysis (DeepScena) and Cluster decomposition-based Anomaly Detection (scCAD), is taking the first steps toward multisample benchmarking to gain insight into the effect of pooling and/or batch correction on the recovery of rare populations (Lei et al., 2023; Xu et al., 2024). Together, these methods justify the inclusion of these approaches in REACH, but they also show why a cancer-specific benchmark is needed: rare malignant cells may not share a single transcriptional program and often require confirmation from multiple lines of evidence (Tirosh and Suva, 2024; Ren et al., 2018).

### 2.2 Single-cell benchmarking

Various mixture control tests have been used to benchmark a number of different scRNA-seq pipelines (Tian et al., 2019). Other studies have provided benchmarks for cell-type classification, batch correction, clustering for tumor types in cancer, automated annotation of tumor cells, and copy number inference (Abdelaal et al., 2019; Tran et al., 2020; Mahalanabis et al., 2022; Chen et al., 2025; Christensen et al., 2023). These studies have established useful practices for benchmarking fixed datasets with clearly defined metrics. However, building on the previous template, the low detection frequency of malignant cells has required prevalence-adjusted methods, null controls, fallback strategies, and ranked metrics (Davis and Goadrich, 2006; Saito and Rehmsmeier, 2015; Johnson and Khoshgoftaar, 2019).

### 2.3 Malignant cell annotation and CNV evidence

Labeling of malignant cells is typically done using the combined results of expression and CNV inference (Gao et al., 2021). Copy number Karyotyping of Tumors (CopyKAT) can delineate copy number and clonal substructures (Gao et al., 2021). Moreover, single-cell Annotation of Tumor and Immune Cells (scATOMIC) assigns cell types to the cells in the tumor microenvironment for all types of cancer (Nofech-Mozes et al., 2023). Here, variant calling has been tested as another way of providing evidence of the presence of a cancer cell (Gas et al., 2022). REACH uses CNV and cancer signature evidence to support label construction rather than to create a separate leaderboard score. In other words, these signals help define high-confidence malignant and background cells, but they are not themselves ranked as competing detection methods. Candidate methods that have failed to provide valid cell-level scores or do not satisfy the required score contract are documented in the method inclusion audit and excluded from the main ranking analyses (Figure 3a).

**FIGURE 3:**
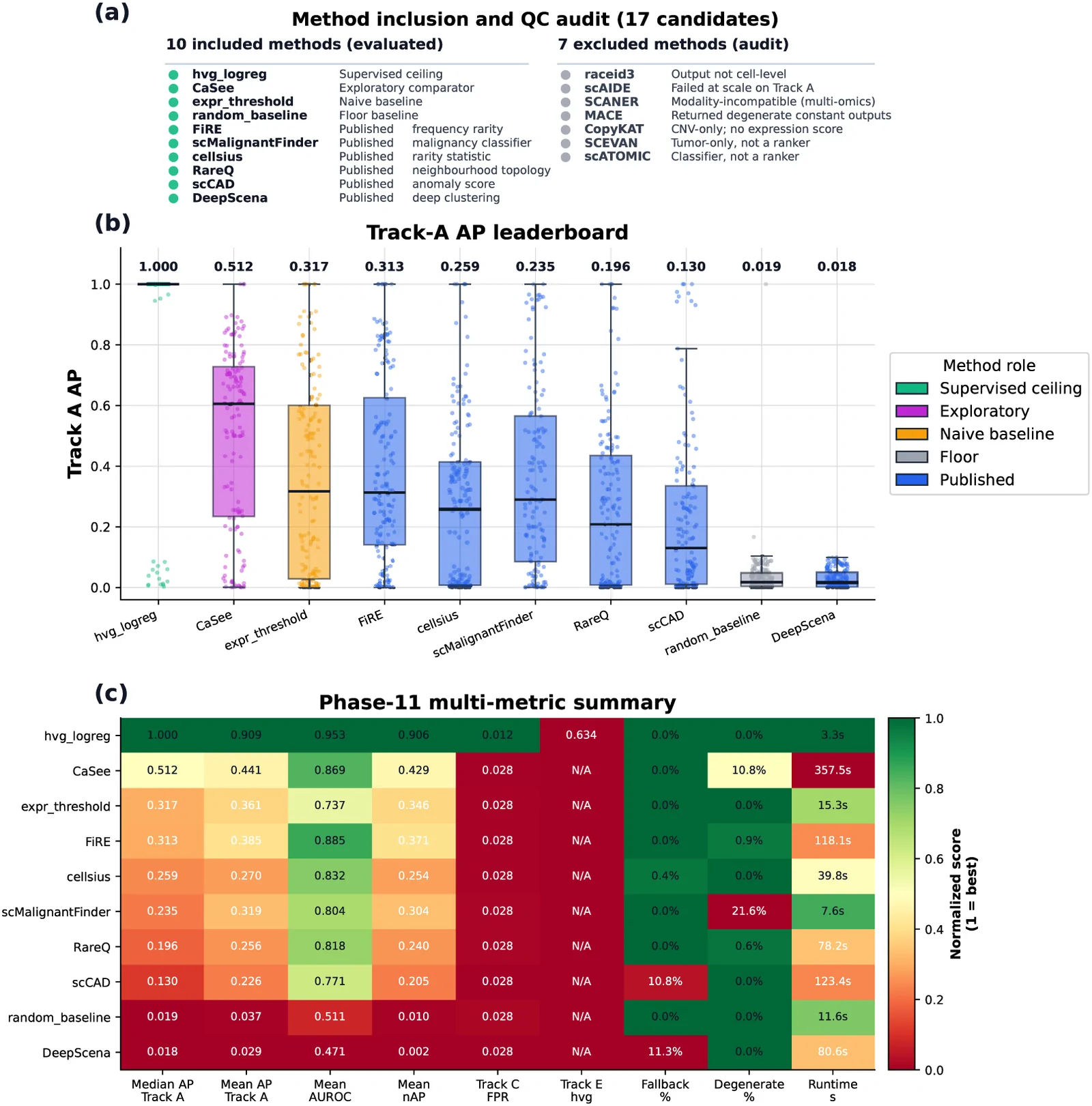
Method audit, leaderboard, and multimetric summary. **(a)** Method inclusion and QC audit. Seventeen candidate methods have been considered. Ten have produced evaluable outputs under the standardized score contract, whereas seven have been excluded because of integration constraints, score contract mismatch, out-of-memory or timeout failures, or degenerate outputs. The audit is part of the benchmark deliverable, not informal curation. **(b)** Track A AP leaderboard. Box-and-strip summaries show fallback-filtered Track A AP by method. The figure separates the supervised ceiling, exploratory comparator, naive baselines, ranked published methods, and random floor. **(c)** Phase 11 multimetric summary heatmap. Min–max scaled values are shown across AP, nAP, AUROC, and MCC at top-k; Track E robustness where applicable; runtime; and null-control behavior. The figure summarizes metric-level performance rather than dataset-by-method behavior.

## 3 Methodology

### 3.1 Task definition and metrics

For benchmark unit u , let $X_{u}\in R^{n_{u}\times p}$ be the log-normalized expression matrix, where $n_{u}$ denotes the number of cells and p the number of genes in unit u . Let $y_{ui}\in \left\{0,1\right\}$ denote the ground truth label of cell i in unit u , where $y_{ui}=1$ indicates a high-confidence malignant (rare-cell) state and $y_{ui}=0$ indicates a background or non-malignant status. A “false-positive” in REACH is, therefore, task-specific: it denotes a cell flagged as rare that is not in the high-confidence malignant set (P_HC), regardless of whether that cell is a biologically valid rare population in its own right (e.g., a rare immune or stromal subtype, an activated or dying cell state).

Here, it should be mentioned that throughout this paper “rare” denotes low-abundance—a population constituting a small fraction of cells in a benchmark unit (Track A tiers T1–T4, 0.01% – 10% )—not a rare biological substate: a transcriptionally distinct condition occupied by a minority of cells within an otherwise homogeneous lineage, such as a drug-tolerant persister, an EMT or partial-EMT intermediate, a transitioning clone, or a novel uncharacterized cell state. The two senses are frequently conflated and impose different requirements on a detector: recovering a low-abundance population requires sensitivity to class imbalance, whereas recovering a rare sub-state additionally requires resolution of the within-lineage transcriptional structure. Tracks A–E measure the first sense only and against an inter-lineage background. Track F relaxes lineage separation but does not measure the second sense directly: it contrasts malignant with normal epithelial cells of the same broad lineage, probes intra-lineage malignancy detection rather than benchmarking rare sub-states within an already-malignant population, and bounds sub-state discovery from above rather than measuring it. Correspondingly, we refer to the evaluated tools as inter-lineage rare malignant cell detectors (equivalently, global tumor anomaly detectors), not as general rare-cell detectors, and the acronym REACH should be read with “rare” carrying the low-abundance sense defined here.

Outlier-based methods such as FiRE and scCAD are designed to surface mathematical outliers and will legitimately detect such TME subpopulations; under REACH’s malignant cell detection contract, these detections count as false positives because they do not solve the interlineage malignant-versus-TME task the benchmark is designed to assess. This convention measures task alignment, not biological validity. A method m returns a continuous cell-level score, as defined in Equation 1:

$$f_{m}\left(X_{u}\right)=s_{u}^{\left(m\right)}\in R^{n_{u}},$$ (1)

where $s_{u}^{\left(m\right)}$ is the vector of predicted scores for all cells in unit u , higher scores indicate a higher likelihood of malignant or rare cell status. REACH evaluates ranking performance rather than thresholded diagnosis. For a ranked list, the standard interpolated average precision (AP) (Equation 2; Davis and Goadrich, 2006; Cormack et al., 2006) is

$$AP\left(m,u\right)=\overset{n_{u}}{\underset{k=1}{\sum }}\left(R_{k}-R_{k-1}\right)\;P_{k},$$ (2)

where $P_{k}$ and $R_{k}$ denote precision and recall after the first k ranked cells, respectively, with $R_{0}=0$ . The primary method summary is the median Track A AP computed across all valid benchmark units (Equation 3):

$$S_{m}=\underset{u\in U_{A,m}^{\text{valid}}}{median}AP\left(m,u\right),$$ (3)

where $U_{A,m}^{\text{valid}}$ denotes the set of Track A benchmark units that remain after fallback-based filtering for method m . Because raw AP depends on class prevalence, REACH additionally reports prevalence-adjusted quantities. Let positive class prevalence

$$\pi_{u}=\frac{1}{n_{u}}\underset{i}{\sum }y_{ui}$$ (4)

in benchmark unit u be defined by Equation 4. AP above chance is defined as $AP\left(m,u\right)-\pi_{u}$ , and the normalized AP (Equation 5) is:

$$nAP\left(m,u\right)=\frac{AP\left(m,u\right)-\pi_{u}}{1-\pi_{u}}.$$ (5)

AUROC is reported as a secondary discrimination metric (Davis and Goadrich, 2006; Saito and Rehmsmeier, 2015). Calibration metrics, including the Brier score and Expected Calibration Error (ECE), are reported only for probability-like outputs and are explicitly marked as not applicable for rank- or anomaly-based scores. F1, precision, recall, balanced accuracy, and Matthews correlation coefficient (MCC) are reported at top k , where k equals the number of positive cells in the corresponding benchmark unit (Johnson and Khoshgoftaar, 2019; Altalhan et al., 2025).

### 3.2 REACH pipeline

REACH is implemented as a 12-phase reproducible pipeline, beginning with environment setup and ending with audited result tables and figures (Figure 1). Phase 0 defines the software environment, directory structure, configuration files, and execution requirements. Phase 1 collects dataset metadata and source files. Phase 2 performs standardized preprocessing, including quality control, normalization, highly variable gene selection, dimensionality reduction, graph construction, and harmonization of cell and gene annotations. Phase 3 assigns confidence tiers to cells by combining source annotations, CNV evidence, cancer signature scores, and neighborhood support.

Phases 4–8.2 generate the benchmark units used for evaluation. Phase 4 creates Track A units for controlled real-cell rarity. Phase 5 creates Track B units for synthetic stress testing. Phase 6 creates Track C background-dominated, null-control units. Phase 7 creates Track D units using natural blood and circulating tumor cell prevalence. Phase 8.1 creates Track E units for supervised label noise analysis by keeping the expression matrix fixed while perturbing labels. Phase 8.2 creates Track F units by masking the global tumor microenvironment contrast to test intra-lineage detection of rare malignant epithelial cells against a normal epithelial background. These phases convert the processed datasets and confidence labels into fixed benchmark units with expression matrices, cell-level labels where applicable, prevalence information, and metadata.

The later phases execute and summarize the benchmark. Phase 9 runs each method through a standardized wrapper and records cell-level scores, runtime information, fidelity status, fallback flags, and degenerate-output flags. Phase 10 collects the prediction outputs across all method–unit combinations. Phase 11 computes ranking metrics, null-control summaries, runtime summaries, and statistical tests. Finally, Phase 12 regenerates the figures and manuscript-level result tables directly from the audited outputs. This phase-based design makes the benchmark traceable because each result can be linked back to its input data, configuration files, and upstream processing contract (Cao et al., 2021).

### 3.3 Datasets and preprocessing

Here, we have processed 10 datasets (Table 2a), such as 8 solid tumor and 2 blood/circulating tumor cells (CTCs). Track A (ranking of solid tumor samples) has been done using the eight solid tumor datasets, whereas Track D (natural prevalence) has been created using the two blood/CTC datasets. Each of the datasets has been processed through a standard AnnData pipeline (Jovic et al., 2022), which includes loading each dataset, standardizing the gene symbol to the normalization standard, obtaining the mitochondrial annotations, performing hard quality control (QC) [minimum genes = 200; minimum unique molecular identifiers (UMIs)= 500; minimum cells = 3], performing the Scrublet doublet detection algorithm (Jovic et al., 2022), where possible, adaptively flagging mitochondrial results, performing total count normalization (to 10,000) with subsequent log1p transformation, providing both raw and normalized layers (Tran et al., 2020), identifying the 2,000 highly variable genes (hvg) (using Seurat V3 or Cell Ranger as fallback) (Tran et al., 2020; Abdelaal et al., 2019), calculating 50 components of principal component analysis (PCA), constructing a neighborhood graph and performing Leiden clustering (with res = 0.5 and seed 42) (Mahalanabis et al., 2022), analyzing the chromosome annotations based on the Genome Reference Consortium Human Build 38 (GRCh38) reference, standardizing the observation columns across all data sets, and finally creating gzipped.h5ad files and Secure Hash Algorithm 256-bit (SHA 256) metadata (Cao et al., 2021).

### 3.4 Multi-arm label construction

All cells acquire a confidence level as an outcome of the source annotation combined with a Python/Scanpy-based tool (infercnvpy CNV inference), area under the curve cell scoring (AUCell) signatures, and the neighborhood support of the individual cells (Chen et al., 2025; Gao et al., 2021; Hetzel et al., 2021; Fa et al., 2026). Two free parameters govern the high-confidence label assignment: the AUCell signature score threshold ( $\tau_{\text{AUCell}}$ , default 0.15) and the kNN neighborhood purity cutoff ( $\tau_{\text{kNN}}$ , default 0.50, applied to the fraction of k=15 PCA nearest neighbors sharing the provisional class). A cell is assigned to the P_HC group when it satisfies all four positive evidence criteria (source positive, aneuploid, AUCell score $\geq \tau_{\text{AUCell}}$ , and neighborhood purity $\geq \tau_{\text{kNN}}$ ) and to the B_HC group when all four-criteria are in the opposite direction. All the datasets have used infercnvpy as the main tool of CNV inference. CopyKAT has not been used as the primary CNV-calling method because it has failed the cell-level score contract and, by design, does not allow a consistent primary wrapper to be applied across different datasets (Chen et al., 2025). As a member of the P_HC, a cell should satisfy all four positive evidence criteria, such as source positive, aneuploid, high signature, and neighborhood support. The same four-criteria should be in the opposite direction for the B_HC group. Here, primary AP has been computed using a subset of P_HC and B_HC cells. Such a design balances two goals, including enough cells for evaluation while maintaining stable and reliable high-confidence labels (Stegle et al., 2015; Abdelaal et al., 2019).

As CNV evidence contributes to high-confidence (HC) label construction, we tested for circularity by evaluating whether the published method ranking is an artifact of CNV-related expression patterns being encoded into the labels. We have rederived HC labels after dropping the CNV criterion entirely using only tissue-of-origin source annotation, AUCell signature scores, and kNN neighborhood purity and recomputed the median AP for every method. The results of this circularity check are reported in Section 7 (Table 9a). Most of the HC label signal comes from CNV. However, this only threatens validity if CNV is also ranked as a competing method. In REACH, CNV is used purely for labeling, not as a scored comparator. Independent validation of the CNV evidence itself against published source annotations is reported in Section 7 (Table 9b).

### 3.5 Label threshold sensitivity

As the high-confidence label assignment depends on two free parameters ( $\tau_{\text{AUCell}}$ and $\tau_{\text{kNN}}$ ), we have performed a sensitivity analysis by varying both across a 3×3 grid: $\tau_{\text{AUCell}}\in \left\{0.10,0.15,0.20\right\}$ and $\tau_{\text{kNN}}\in \left\{0.40,0.50,0.60\right\}$ , yielding nine threshold combinations per dataset. Phase-1 infercnvpy CNV scores were held fixed so that only the threshold-dependent criteria were varied. For each combination, we have rederived the P_HC and B_HC label sets, recomputed the per method Track A median AP, and measured the Spearman rank correlation ρ between the rederived-label ranking and the published (default-threshold) ranking (Table 1).

**TABLE 1:** Sensitivity analyses.

Dataset	$n_{P\text{\_}HC}$	$n_{B\text{\_}HC}$	Spearman ρ	p -value
(a)				
crc_lee	204	0	0.939	$5.5\times 10^{-5}$
hcc_wei	210	0	0.879	$8.1\times 10^{-4}$
bcc_yost	473	0	0.818	$3.8\times 10^{-3}$
pdac_peng	452	0	0.758	$1.1\times 10^{-2}$
luad_laughney	593	0	0.685	$2.9\times 10^{-2}$
rcc_multi	180	0	—	Excluded (insufficient ranking overlap)
ov_izar_tirosh	40/3/1	0	—	Excluded (threshold-sensitive P_HC count)

Method	Track A rank	Track B rank	Δ rank
(b)			
hvg_logreg	1	1	0
CaSee	2	6	−4
FiRE	3	7	−4
expr_threshold	4	9	−5
scMalignantFinder	5	10	−5
CellSIUS	6	3	+3
RareQ	7	2	+5
scCAD	8	4	+4
random_baseline	9	8	+1
DeepScena	10	5	+5
Spearman ρ=0.042 , p=0.907 (Track A vs. Track B ranking)			

(a) Label threshold sensitivity. Spearman rank correlation ρ between the rederived-label method ranking and the published (default $\tau_{\text{AUCell}}=0.15$ , $\tau_{\text{kNN}}=0.50$ ) ranking, across nine threshold combinations (AUCell ∈{0.10,0.15,0.20} × kNN ∈{0.40,0.50,0.60} ) with CNV scores held fixed. On the five valid datasets, the P_HC label set was invariant across all nine combinations, so ρ is constant within each dataset; the range [0.685,0.939] reflects cross-dataset variation. Two datasets are excluded. (b) Track A vs. Track B method rank comparison. Method-level Spearman ρ=0.042 (p=0.907) across the 10 methods. Only the supervised hvg_logreg ceiling retains its rank (1 → 1); all ranked published methods reorder substantially, indicating that synthetic Splatter stress captures different method properties than real-cell spike-ins. Source: data/results/sens2_sens3_extract.txt.

On the five datasets yielding a valid ρ , the P_HC label set has been invariant across all nine threshold combinations, so the within-dataset ρ has been constant, and the published ranking has been exactly reproduced. The cross-dataset ρ range has been [0.685,0.939] (all p<0.05 ): crc_lee 0.939, hcc_wei 0.879, bcc_yost 0.818, pdac_peng 0.758, and luad_laughney 0.685. Two datasets have been excluded: rcc_multi has produced a constant P_HC set (n=180) but insufficient ranking overlap for a valid ρ , and ov_izar_tirosh has been the sole dataset where the P_HC count has varied with the threshold ( n=40,3,1 across the tested grid), reflecting threshold-sensitive labels that have prevented a stable ρ . The qualitative method ranking is therefore stable across plausible threshold choices on the five datasets where the analysis is valid; the threshold sensitivity of ov_izar_tirosh is detailed in Section 7.

We also note a structural property of the label design: on all seven datasets and all nine threshold combinations, no cell has satisfied the full four-criteria intersection for the B_HC group $\left(n_{B\text{\_}HC}=0\right)$ . Track A background cells are therefore drawn from the broader source-negative, diploid, low-signature pool rather than from a strict B_HC set. This does not affect the validity of the Track A ranking—which depends on the P_HC positives being correctly labeled—but it means the B_HC arm does not, in the current snapshot, produce a usable symmetric high-confidence background set. Future versions of REACH should relax or restructure the B_HC criteria to enable symmetric high-confidence labeling.

### 3.6 Evaluation tracks

We use six tracks in the REACH pipeline (Figure 2; Table 2): five primary tracks (A–E) built on the interlineage malignant-versus-TME contrast, plus one intralineage diagnostic track (Track F) that masks the global TME contrast to test within-lineage malignant detection. Track A samples real P_HC cells into a mock background at four prevalence tiers (T1: 5– 10% , T2: 1– 5% , T3: 0.1– 1% , and T4: 0.01– 0.1% ), targeting approximately 2,000 cells per unit with <20% duplicates (Johnson and Khoshgoftaar, 2019; Altalhan et al., 2025). Track B uses Splatter simulations fitted to each real dataset and audited for library size, dropout, and differential-expression overlap (Cao et al., 2021). Track B is deliberately a secondary stress test, not a primary endpoint: the method-level Spearman rank correlation between Tracks A and B is ρ=0.042 (p=0.907) , statistically indistinguishable from zero, with large rank reordering across methods (Table 1). Only the supervised hvg_logreg ceiling retains its rank (1→1) . The near-zero ρ is the intended signal—a synthetic stress test that has perfectly replicated real-cell rankings would be redundant—so Track B is reported separately, not blended into the primary Track A leaderboard. Track C uses background-only null samples (Torre et al., 2018). Track D uses the natural prevalence of mm_ledergor and breast_ctc_szczerba (Johnson and Khoshgoftaar, 2019; Altalhan et al., 2025). Track E fixes Track A expression matrices and measures four corrupted-label conditions (10% symmetric flip, 20% symmetric flip, 30% positive → background, and 30% background → positive); it is interpreted as a supervised hvg_logreg ceiling diagnostic only (Cao et al., 2021).

**TABLE 2:** Benchmark datasets and evaluation tracks.

Dataset id	Cancer type	Platform	Tracks	Final cells
(a)				
hnscc_puram	Head and neck SCC	Smart-seq2	A/B/C/E	5,902
ov_izar_tirosh	Ovarian cancer ascites	10× Chromium	A/B/C/E	9,482
hcc_wei	Hepatocellular carcinoma	10× Chromium	A/B/C/E	19,382
luad_laughney	Lung adenocarcinoma	10× Chromium	A/B/C/E	33,782
rcc_multi	Renal cell carcinoma	10× Chromium	A/B/C/E	33,574
pdac_peng	Pancreatic ductal adenocarcinoma	10× Chromium	A/B/C/E	123,488
crc_lee	Colorectal cancer	10× Chromium	A/B/C/E	55,551
bcc_yost	Basal cell carcinoma	10× Chromium	A/B/C/E	∼47,000
mm_ledergor	Multiple myeloma	10× Chromium	D	31,181
breast_ctc_szczerba	Breast cancer CTCs	Smart-seq2	D	357

Track	Purpose	Units	Primary interpretation
(b)			
A	Controlled real P_HC spike-ins into B_HC background	160	Primary ranked endpoint
B	Synthetic splatter stress with realism audits	120	Secondary stress test
C	Background-dominated null controls	160	Specificity pass/fail diagnostic
D	Natural blood/CTC prevalence	30	Natural prevalence supplement
E	Track A expression with corrupted labels	640	Supervised label noise analysis
F	Intra-lineage: P_HC malignant epithelial spike-ins into normal epithelial B_HC (crc_lee only; 0.1%, 0.5%, and 1.0% prevalence; 5 replicates each)	15	Intra-lineage diagnostic; not blended into the primary leaderboard
Total (primary, Tracks A–E)		1,110	
Track F (diagnostic, reported separately)		15	

(a) Datasets. Final cell counts follow the Phase 1–12 report and are internally consistent with the tier count table. Gene Expression Omnibus (GEO) accessions are listed in the section Data and Code Availability. (b) Audited unit counts per method per track. The primary benchmark (Tracks A–E) contains 1,110 units, yielding 11,100 method–unit evaluations across the ten included methods. Track F is reported separately as a diagnostic benchmark.

Track F signifies intralineage malignant detection. In direct response to the within-lineage benchmarking requirement, Track F masks the global TME contrast that defines Tracks A–E and tests whether methods can recover rare malignant epithelial cells hidden among normal epithelial cells of the same tissue origin. The background pool is restricted to normal-diploid epithelial cells (tissue_origin == “Normal,” cell_type == “Epithelial cells”); positives are malignant epithelial cells (tissue_origin == “Tumor,” high confidence from Track A). Units are generated at three prevalences (0.1%, 0.5%, and 1.0%) with 5 replicates each (15 units total); unit sizes are adaptive (2,000 cells at 0.1% to preserve the ultra-rare signal with ≥2 positives and 400 cells at 0.5% and 1.0% to keep background duplication below the 20% design cap). All 10 methods have been evaluated (150/150 predictions completed). Track F is constructed from the crc_lee (colorectal cancer) dataset only because it is the sole REACH dataset carrying both epithelial cell-type annotations and normal/tumor tissue origin labels; the other nine datasets lack the required normal epithelial reference even via marker-based fallback (epithelial cell adhesion molecule ${\left(EPCAM\right)}^{+}$ ∩ low-CNV ∩ source-negative yields 0–70 cells). Track F is a diagnostic track and is not blended into the primary Track A leaderboard.

The intra-lineage principle—that inter-lineage leaderboards cannot be extrapolated to within-lineage malignant detection—is confirmed by the Track F results reported in Section 5.2 (nine of ten methods collapse to median AP ≤0.009 at 0.1% prevalence; only the colorectal-cancer-specific scMalignantFinder retains power).

### 3.7 Method execution, score contracts, and quality control

All methods run under a common wrapper contract (input h5ad, output directory, and config) and produce a predictions.csv file of cell-level scores plus a runmeta.json file recording runtime, memory, version, success/failure status, fidelity status, and degenerate-output flags. Scores are continuous and cell-level, with higher scores indicating stronger evidence of malignancy or rarity (Märtens et al., 2023; Xu et al., 2024).

REACH assesses ten methods in five interpretive categories (Table 3): a supervised in-sample ceiling (hvg-logreg), an exploratory cancer/normal comparator (CaSee), six ranked published interlineage detectors (FiRE, DeepScena, RareQ, CellSIUS, scCAD, and scMalignantFinder), and two naive baselines (expr_threshold and random_baseline) (Wegmann et al., 2019; Xu et al., 2024; Yu et al., 2025). Methods failing the score contract or resource limits are recorded in the inclusion audit (Figure 3) and excluded from ranking.

**TABLE 3:** Included methods and interpretive roles.

Method	Category	Output type	Role
random_baseline	Naive	Uniform random score	Floor baseline
expr_threshold	Naive	Total UMI/signature expression rank	Naive biological baseline
hvg_logreg	Supervised	Logistic probability on 2,000 HVGs	In-sample ceiling
FiRE	Ranked	Frequency-based rarity score	Published comparator
DeepScena	Ranked	Deep clustering/SSL score	Published comparator
RareQ	Ranked	Neighborhood rarity score	Published comparator
CellSIUS	Ranked	Rarity statistic	Published comparator
scCAD	Ranked	Anomaly score	Published comparator
scMalignantFinder	Ranked	Malignancy probability	Published/proxy comparator
CaSee	Exploratory	Autoencoder + isolation ${\text{Forest}}^{†}$	Exploratory comparator (cancer/normal scorer)

† CaSee ran as a faithful CPU recreation of the original autoencoder, not the original GPU-dependent code.

Only the “ranked published” category participates in the primary leaderboard; the supervised ceiling and exploratory comparator are reported separately. This categorization is a scoping convention, not a performance judgment: CaSee outperforms several ranked published methods on Track A but is reported separately because it is a cancer/normal scorer rather than a dedicated rare-cell detector (see Section 5.2 for the ceiling interpretation). For methods with degenerate outputs, both all-units and degenerate-excluded median AP values are reported.

Fallback scores (random scores after timeout or memory failure) are filtered from AP aggregation. Degenerate scores (constant or near-constant outputs) are retained, flagged, and reported. The audited snapshot contains 249 fallback units (DeepScena 125, scCAD 120, and CellSIUS 4) and 377 degenerate units (scMalignantFinder 240, CaSee 120, FiRE 10, and RareQ 7).

### 3.8 Reproducible extension protocol

REACH is extensible. If users want to add a new method, then they have to implement the wrapper that follows the standard REACH run contract and register that method in the method registry. Basic metadata and a smoke test that identifies it can run correctly must be included within each method before being added to the overall benchmark. Output from the system is designed to be very simple: a score (at cell-level), an optional predicted label, runtime metadata, fidelity and fallback, or degenerate flags.

We can also add new datasets, which involves creating a dataset-registry entry, raw-data loader, source annotation mapper, signature configuration, preprocessing outputs, and the corresponding Track A–F benchmark units. Likewise, to add a new evaluation track or metric, one needs to provide updates on the appropriate track generator, evaluator (wherever it is defined), aggregation tables, and figure generation scripts (Everingham et al., 2010; Abdelaal et al., 2019; Tran et al., 2020).

### 3.9 Computational environment

All computational analyses have been executed on a CPU-only node (AMD EPYC 7B12, 62 GiB RAM, Debian 13, and Python 3.13.5 and R 4.5.0); DeepScena has been run on a Google Colab Tesla T4 GPU. Runtimes are comparable across CPU methods; DeepScena’s GPU runtime is reported but not directly comparable. The full environment capture and per-method resource settings are documented in data/results/environment/environment_capture.txt, and the wrapper configurations are provided in the public repository.

## 4 Experiments

### 4.1 Benchmark scale

Across ten methods, REACH evaluates 1,125 benchmark units per method, resulting in 11,250 method–unit evaluations (Abdelaal et al., 2019; Abdelaal et al., 2025). The primary leaderboard displays median AP on Track A after method-specific fallback filtering. The global and pairwise rank tests use the 140-unit common subset of Track A retained after method-specific fallback filtering, with 2,000 bootstrap resamples calculated for rank intervals (Cao et al., 2021).

### 4.2 Statistical testing

A Friedman test with Iman–Davenport correction has been used to assess global differences in per-unit Track A AP across methods (Gorodkin, 2004). Wilcoxon signed-rank tests against the top published method (FiRE) have been performed for each pairwise comparison, with Benjamini–Hochberg false discovery rate (FDR) correction. Because statistical significance alone can overstate practical relevance under large sample sizes, we report two complementary effect size measures for every pairwise comparison: the mean AP difference ( Δ mean AP) and Cliff’s δ , a non-parametric effect size robust to non-normal score distributions (Romano et al., 2006). The full 10×10 pairwise Cliff’s δ matrix is reported in Table 7, and selected pairwise comparisons against FiRE are reported in Table 6. Rank uncertainty estimates are derived from 2,000 bootstrap resamples (rank_ci.csv). Rarity sensitivity is assessed by regressing nAP against $\log_{10}\pi_{u}$ (Saito and Rehmsmeier, 2015; Davis and Goadrich, 2006).

## 5 Results

### 5.1 Primary Track A leaderboard

The primary leaderboard is grouped by the median AP following the fallback filter (Table 4; Figure 3b). The median AP of 1.00 for hvg-logreg indicates that many high-confidence malignant and background labels contain gene expression differences that can be recovered by a supervised model (Stegle et al., 2015; Ren et al., 2018).

**TABLE 4:** Primary Track A performance from statistical_ranking.csv.

Role rank	Method	Median AP	Mean AP	AUROC	Runtime (s)	Role
–	hvg_logreg	**1.000**	0.909	0.953	3.3	Supervised ceiling
–	CaSee	0.512	0.441	0.869	357.5	Exploratory
–	expr_threshold	0.317	0.361	0.737	15.3	Naive baseline
1	FiRE	0.313	0.385	0.885	118.1	Published
2	CellSIUS	0.259	0.270	0.832	39.8	Published
3	scMalignantFinder	0.235	0.319	0.804	7.6	Published/proxy
4	RareQ	0.196	0.256	0.818	78.2	Published
5	scCAD	0.130	0.226	0.771	123.4	Published
–	random_baseline	0.019	0.037	0.511	11.6	Floor baseline
6	DeepScena	0.018	0.029	0.471	80.6‡	Published

*Methods with degenerate Track A units: CaSee median AP, 0.512 (all 160 units)/0.606 (140 units excl. 20 degenerate); scMalignantFinder median AP, 0.235 (all 160)/0.290 (140 excl. 20 degenerate); and RareQ median AP, 0.196 (all 160)/0.208 (159 excl. 1 degenerate). Runtimes are mean Track A wall-clock times on the computational environment (Section 3.9); DeepScena (^‡^) has been run on a Google Colab Tesla T4 GPU, and its runtime is not directly comparable with the CPU-only methods.

Runtime is the mean Track A wall-clock time. Only the “ranked published” category (role ranks 1–6) participates in the primary leaderboard; the supervised ceiling, exploratory comparator, naive baseline, and floor baseline are reported for reference but are not assigned leaderboard ranks. The exploratory comparator CaSee is excluded from the ranked category because it is a cancer/normal cell scoring framework rather than a dedicated rare-cell detector, produces degenerate outputs on 20/160 (12.5%) Track A units, and runs as a faithful CPU recreation of the original autoencoder; both its all-units and degenerate-excluded median AP values are reported in the ${\text{footnote}}^{*}$ . Ranks in this table are interlineage ranks obtained against a background of immune, stromal, and normal epithelial cells (Table 5). They do not transfer to an intralineage setting. Bold values indicate the top-performing method within each metric or evaluation role.

CaSee is ranked as the second highest median AP (0.512 on all 160 Track A units and 0.606 after excluding 20 degenerate units), but it is reported as an exploratory comparator rather than as a primary ranked published detector. The rationale for this categorization is stated in the method inclusion section and rests on four converging reasons: (i) CaSee is a cancer/normal cell scoring framework rather than a dedicated rare-cell detector, so it belongs to a different method class from the ranked published category; (ii) CaSee produces constant score degenerate outputs on 20 of 160 (12.5%) Track A units, so its median AP mixes real rankings with degenerate outputs; (iii) CaSee has been run as a faithful CPU recreation of the original autoencoder rather than the original GPU-dependent code; and (iv) CaSee is the slowest method in the benchmark (mean execution time 357.5 s, approximately 3× FiRE). CaSee nonetheless significantly outperforms FiRE on the 140 common non-degenerate units (Wilcoxon $p=3.96\times 10^{-8}$ , Cliff’s δ=0.245 ), and this exploratory comparison is reported separately under the exploratory versus published category rather than the primary ranked leaderboard (Yuan et al., 2022). FiRE has the highest median AP (0.313) among the ranked published comparators. The naive expr_threshold baseline is slightly larger than FiRE’s median AP (0.317) but is not presented as a published detector. The reported median of the naive expr_threshold is greater than that of FiRE and is to be interpreted as a caution that a naive approach to the expression burden may be competitive in some cancers (i.e., naive threshold expressed in earlier reports) (Torre et al., 2018).

### 5.2 Interpretation of the supervised ceiling

The near-perfect Track A performance of the hvg-logreg ceiling (median AP = 1.000) could reflect genuine biological separability between high-confidence malignant and background cells or the conservative four-criteria label construction that selects only cells with unambiguous multi-evidence support. Three independent lines of evidence indicate that biological separability is the primary driver, with label conservatism playing a secondary role.

First, the ceiling is not uniformly near-perfect across datasets. On seven of eight datasets, hvg-logreg achieves median AP = 1.000 (131 of 160 units at AP = 1.000); on ov_izar_tirosh, it collapses to median AP = 0.054 (minimum 0.003), where several unsupervised methods outperform it (expr_threshold 0.659 and FiRE 0.592). If label conservatism is the sole driver, the ceiling would be uniformly high; the dataset-specific collapse shows that it tracks the underlying biological separability.

Second, the ceiling collapses on the intralineage Track F: hvg-logreg drops from median AP = 1.000 on Track A to 0.001 at 0.1% prevalence (Δ=−0.999) , outputting constant 0.5 scores because of the absence of the interlineage contrast on which training has been done (Table 5). This drop cannot be attributed to label conservatism—the labels are equally conservative on both tracks—and reflects the removal of the interlineage contrast.

**TABLE 5:** Track F intralineage leaderboard.

Method	Category (interpretive role)	Track F median AP		
		0.1%	0.5%	1.0%
scMalignantFinder	Ranked published	1.000	1.000	0.917
expr_threshold	Naive baseline	0.009	0.062	0.154
FiRE	Ranked published	0.005	0.022	0.041
CaSee	Exploratory	0.003	0.049	0.063
random_baseline	Floor baseline	0.002	0.008	0.016
CellSIUS	Ranked published	0.001	0.007	0.008
RareQ	Ranked published	0.001	0.005	0.010
scCAD	Ranked published	0.001	0.005	0.010
DeepScena	Ranked published	0.001	0.005	0.010
hvg_logreg (ceiling)	Supervised ceiling	0.001	0.005	0.010

Median AP per method at three spike-in prevalences (0.1%, 0.5%, and 1.0%) on the crc_lee dataset, where the background is restricted to normal-diploid epithelial cells of the same tissue origin as the malignant positives (global TME removed). Five replicates per prevalence tier (15 units per method; 150/150 predictions completed; DeepScena on a Google Colab Tesla T4 GPU). Methods are sorted by 0.1% median AP (the ultra-rare regime). The supervised hvg_logreg ceiling—perfect (1.000) on interlineage Track A—collapses to the chance floor on Track F at every prevalence because it has been trained on the interlineage malignant-versus-TME contrast and outputs constant 0.5 scores when that contrast is masked. Source: data/results/track_f/track_f_leaderboard.csv.

Third, under Track E label noise conditions, the hvg-logreg mean AP degrades from 0.909 (clean labels) to 0.460–0.534 under 30% label flips, confirming that the ceiling responds to label quality rather than being an artifact of conservative selection. We therefore interpret the hvg-logreg ceiling as a probe of interlineage malignant-versus-TME separability recoverable when label information is available, modulated by a secondary contribution from conservative label construction. The conservative assignment contributes to the ceiling’s cleanliness but does not manufacture separability where none exists. The gap between the ceiling and unsupervised methods on Track A thus reflects a real capability gap, not an artifact of label design.

The Track F collapse is not unique to the ceiling—it is the dominant pattern across the full method panel (Table 7). At 0.1% prevalence on Track F, nine of ten methods fall to median AP ≤ 0.009, with the supervised ceiling itself dropping from 1.000 (Track A) to 0.001 (Δ=−0.999) ; every unsupervised method drops by a comparable margin (CaSee 0.512 → 0.003, FiRE 0.313 → 0.005, expr_threshold 0.317 → 0.009, CellSIUS 0.259 → 0.001, RareQ 0.196 → 0.001, scCAD 0.130 → 0.001, and DeepScena 0.018 → 0.001). The sole exception is scMalignantFinder, which rises from 0.235 (Track A) to 1.000 (Track F, 0.1%) and stays dominant at 0.5% (1.000) and 1.0% (0.917)—but this result is colorectal-cancer-specific (Track A AP ranges from 0.015 on hnscc_puram to 0.943 on crc_lee) and must not be generalized (see Section 7). The practical conclusion states that methods whose Track A ranking rests on the inter-lineage malignant-versus-TME contrast lose essentially all discriminative power once that contrast is masked. However, the inter-lineage ceiling is trivial as a detection target. The 1.0 ceiling reflects massive inter-lineage expression variance; a lineage-masking scorer (EPCAM/keratin vs. immune/stromal markers) would approach this bound. The ceiling is therefore an inter-lineage separability reference, not a target that unsupervised rare-cell finders should reach. REACH’s unsupervised methods do not approach 1.0—expr_threshold 0.317, CaSee 0.512, scCAD 0.130, and DeepScena 0.018 (Table 4)—because they are rarity/density detectors rather than lineage separators; the ceiling’s collapse to 0.001 on Track F confirms that 1.0 has been the inter-lineage variance. The 0.7 AP gap is framed as “unsupervised rarity detectors do not fully exploit the inter-lineage signal a supervised method trivially captures,” not as failure at an easy task. A pure lineage-marker baseline is not yet included (see Section 7).

The full Track F leaderboard across all three prevalence tiers is reported in Table 5. This is the “0.1% tumor spike-in within 99.9% normal epithelial cells” design at the 0.1% tier, a unit of 2,000 cells contains ≈2 malignant epithelial positives hidden among ≈1,998 normal epithelial background cells, with the global TME (immune, stromal, and normal non-epithelial) removed. Only scMalignantFinder (a pretrained pan-cancer classifier) solves this regime; every other method, including the perfect-on-Track-A supervised ceiling, sits at the chance floor.

The prevalence-dependent pattern in Table 5 confirms this: only scMalignantFinder solves the 0.1% regime (AP = 1.000), whereas every other method and the supervised ceiling remain ≤ 0.063 at every prevalence. The naive expr_threshold baseline climbs with prevalence (0.009 → 0.062 → 0.154)—mean-expression ranking recovers malignant cells once the spike-in is large enough to shift the transcriptome mean—but stays near chance at the 0.1% ultra-rare tier that defines “rare-cell discovery.” Track F thus confirms that transcriptomic within-lineage rare-cell discovery at 0.1% prevalence is not solved by any general-purpose unsupervised rarity detector in the REACH panel.

The failure mode of the cluster-decomposition methods deserves comment because scCAD is designed to isolate rare populations by iteratively decomposing clusters and scoring the resulting subclusters for anomaly, and normal versus malignant epithelial cells should, in principle, be separable under such a procedure. Two observations constrain the explanation. First, scCAD’s Track F ranking is not merely uninformative but inverted: its mean AUROC is 0.365 (median 0.342), with 13 of 15 units below 0.5 (one-sided binomial p=0.0037 ), against a mean AUROC of 0.719 on prevalence-matched crc lee Track A units where scCAD has executed faithfully on all 20 units. A method that has simply lost the signal would sit near 0.5; a value significantly below it means the normal epithelial background is being ranked systematically above the malignant positives. Second, the scores that these methods emit are piecewise constant over clusters rather than per-cell: across 2,000-cell units, scCAD returns a median of 30 distinct values, DeepScena 13, CellSIUS 10, and RareQ 4, whereas the per-cell scorers return an essentially unique value per-cell (CaSee 2,000, FiRE 1,996, and expr_threshold 1,938). Once positives are absorbed into a larger subcluster, no within-cluster ordering can recover them, and scCAD accordingly returns an AP exactly equal to the prevalence-defined chance value for 10 of the 15 Track F units.

We therefore offer the following putative account: cluster-level scoring defines the anomalous unit as a small, transcriptionally coherent cluster relative to the rest of the sample. In an interlineage background, this coincides with the benchmark’s target because the malignant epithelial cells are both rare and the most transcriptionally distinct group present—which is why these methods rank respectably on Track A. In the Track F background of a normal epithelium, the two criteria come apart: the malignant spike-in is rare but transcriptionally adjacent to the background, whereas the most distinct minority structures produced by the decomposition are subpopulations of the normal epithelium itself. A cluster-level scorer will then preferentially nominate a normal epithelial structure and rank the malignant positives below it, which is what the subchance AUROC records. The mean AUROC pattern across method families is consistent with this account: cluster-based methods average below 0.5 on Track F (scCAD 0.365, CellSIUS 0.319, RareQ 0.390, and DeepScena 0.433), whereas per-cell scorers average above it (FiRE 0.705, CaSee 0.789, and expr_threshold 0.843), although the separation is a family-level tendency rather than a universal per-unit result. Confirming which cells populate the nominated clusters would require a per-unit membership analysis against subtype annotation, which we have not performed; we therefore state the cluster-identity claim as proposed, not established.

### 5.3 Multimetric summary and dataset heterogeneity

Heatmaps of Phase 11 (Figure 3c) reveal trade-offs between methods for all APs, nAPs, AUROC, and MCC at the top of each list (and sometimes Track E robustness), as well as for runtime and null-control behavior for all methods tested. There is no overall winner in all categories (Wang et al., 2022; Bren et al., 2022). The best datasets vary (Table 6): CaSee is the best on four datasets (bcc yost, pdac peng, luad laughney, and hcc wei); expr_threshold is the best on two datasets (hnscc puram and ov izar tirosh). Conversely, CellSIUS is the best on one dataset (rcc multi), whereas scMalignantFinder is the best on one dataset (crc lee). This variability justifies the reporting method performance according to the context (tumor type) rather than a “best method” story (Peng et al., 2019; Qian et al., 2020; Tirosh and Suva, 2024).

**TABLE 6:** Dataset-level performance and statistical comparisons.

Dataset	Best overall	AP	Best non-ceiling	AP
(a)				
bcc_yost	hvg_logreg	1.0000	CaSee	0.6079
hnscc_puram	hvg_logreg	1.0000	expr_threshold	0.7872
pdac_peng	hvg_logreg	1.0000	CaSee	0.4488
rcc_multi	hvg_logreg	1.0000	CellSIUS	0.1931
crc_lee	hvg_logreg	0.9998	scMalignantFinder	0.8373
luad_laughney	hvg_logreg	0.9983	CaSee	0.5378
hcc_wei	hvg_logreg	0.9948	CaSee	0.4908
ov_izar_tirosh	expr_threshold	0.5717	expr_threshold	0.5717

Comparator	Δ mean AP	Wilcoxon p	BH-FDR	Cliff’s δ
(b)				
DeepScena	0.3320	$1.10\times 10^{-24}$	$1.66\times 10^{-23}$	0.7539
random_baseline	0.3226	$2.52\times 10^{-22}$	$8.11\times 10^{-22}$	0.7271
CaSee	−0.1395	$3.96\times 10^{-8}$	$6.14\times 10^{-8}$	−0.2451
scCAD	0.1350	$1.56\times 10^{-4}$	$2.34\times 10^{-4}$	0.2886
RareQ	0.1161	$1.77\times 10^{-3}$	$2.49\times 10^{-3}$	0.2457
CellSIUS	0.0978	$3.06\times 10^{-3}$	$4.04\times 10^{-3}$	0.1930
scMalignantFinder	0.0453	0.812	0.831	0.1206
expr_threshold	0.0304	0.836	0.836	0.0816

(a) Best overall and best non-ceiling method by dataset, copied from best_model_per_dataset.csv. The non-ceiling method includes exploratory and naive comparators. (b) Selected pairwise comparisons on Track A AP from pairwise_tests.csv, computed on the 140 common unit subset. Positive Δ means that FiRE has higher mean AP than the comparator; negative Δ means that the comparator has a higher mean AP. Cliff’s δ uses the same sign convention. The full 10×10 pairwise Cliff’s δ matrix for all method pairs is reported in Table 7; the magnitude bands follow the classifications proposed by Romano et al. (2006).

### 5.4 Statistical significance and rank uncertainty

The overall rank tests have been conducted on the 140 common unit Track A subset. These tests have showed significant differences across methods: Friedman $\chi^{2}=695.05$ , p<0.001 ; Iman–Davenport F=171.01 , p<0.001 (Davis and Goadrich, 2006; Saito and Rehmsmeier, 2015). The results of the pairwise Wilcoxon tests relative to FiRE (Table 6) suggest that FiRE has significantly better performance than random baseline, DeepScena, scCAD, RareQ, and CellSIUS ( p<0.05 after Benjamini–Hochberg FDR correction). There are no significant differences between scMalignantFinder and expr_threshold methods when compared with FiRE. In our exploratory comparison, CaSee is better than FiRE ( Δ mean AP = −0.140 , Benjamini–Hochberg (BH)-FDR = $6.1\times 10^{-8}$ ; Cliff’s δ=−0.245 ); this is reported under the exploratory versus published category, not under the primary ranked method category (Yuan et al., 2022).

The bootstrap intervals for ranks are stable, whereas the hvg-logreg and CaSee methods are ranked 1 and 2, respectively, for every resample of 2,000. Conversely, FiRE is observed to have a median rank of 3 (with a 95% bootstrap rank interval of [3, 4] derived from 2,000 resamples). expr_threshold, scMalignantFinder, CellSIUS, RareQ, and scCAD fall into an overlapping middle band (Figure 4a). The mean within unit rank for each method, shown in Figure 4b, separates the strongest methods from the overlapping middle group and is consistent with the bootstrap rank intervals (Gorodkin, 2004).

**FIGURE 4:**
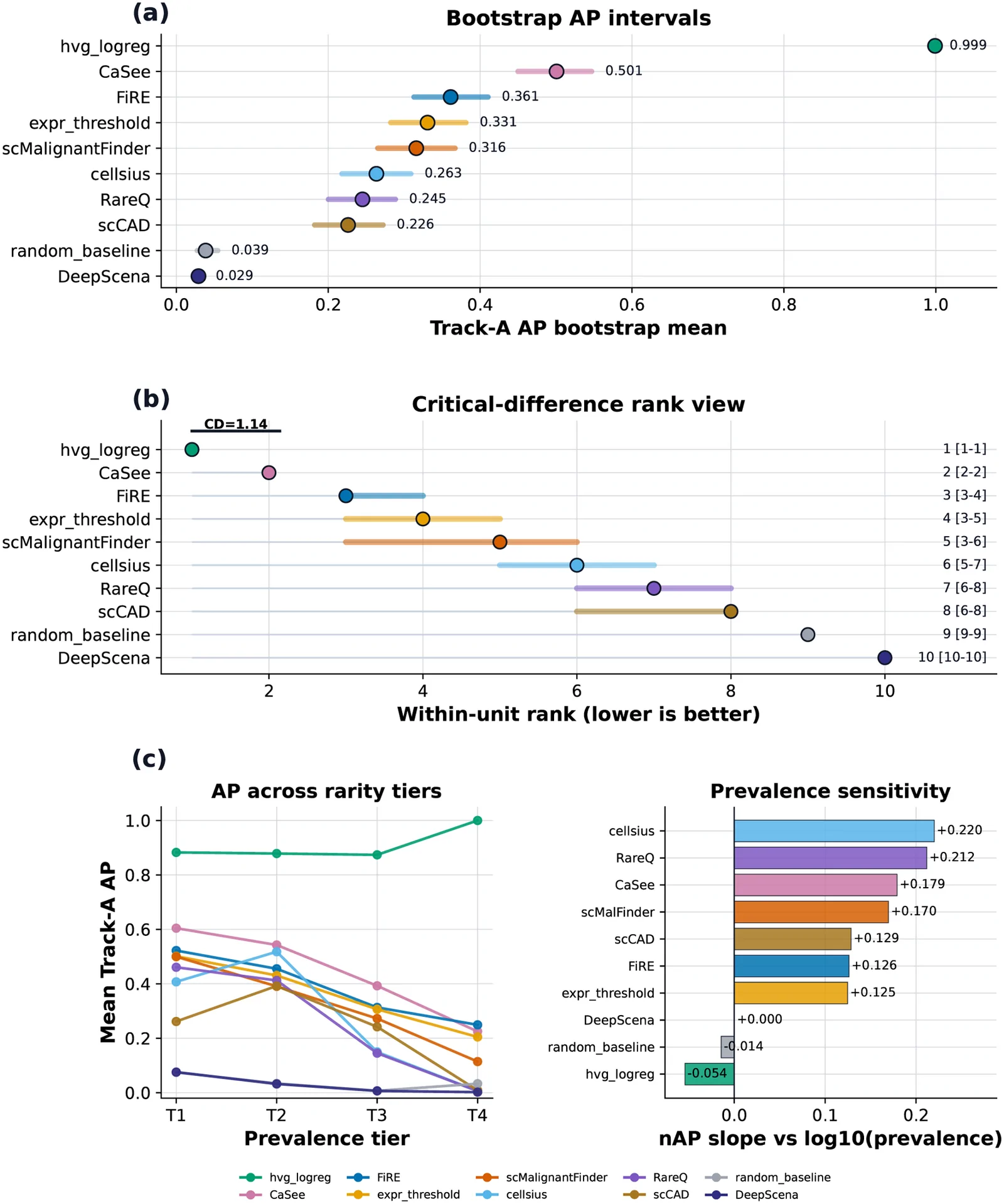
Rank uncertainty, critical difference, and rarity sensitivity. **(a)** Bootstrap rank confidence intervals. Ranks are computed from Track A AP over 2,000 unit level bootstrap resamples (rank_ci.csv). A lower rank is better. **(b)** Critical difference rank view. It shows the mean within unit ranks on the common Track A subset, with the Nemenyi critical difference bar (CD  ≈ 1.14 , k=10 , N=140 , α=0.05 ). **(c)** Rarity sensitivity. It depicts AP and normalized AP across Track A prevalence tiers T1–T4. Several methods degrade sharply in ultra-rare units, whereas the supervised ceiling remains near 1 across tiers.

To complement the significance tests with practical relevance, we have computed the full pairwise Cliff’s δ effect size matrix for all 10 methods on per-unit Track A AP (Table 7). Cliff’s δ is a non-parametric effect size estimating $P\left(X_{A}>X_{B}\right)-P\left(X_{B}>X_{A}\right)$ on paired per-unit measurements and is therefore well suited to benchmark comparisons where normality cannot be assumed. We adopt the conventional magnitude bands (Romano et al., 2006): |δ|<0.147 negligible, 0.147≤|δ|<0.330 small, 0.330≤|δ|<0.474 medium, and |δ|≥0.474 large. Across the 45 unique method pairs, 17 are large, 9 medium, 9 small, and 10 negligible.

**TABLE 7:** Pairwise Cliff’s δ effect size matrix on per-unit Track A AP. Positive δ (row vs. column) means that the row method has higher per-unit AP than the column method. Magnitude bands (Romano et al., 2006): |δ|<0.147 negligible, 0.147–0.330 small, 0.330–0.474 medium, and ≥0.474 large. Across the 45 unique pairs: 17 large, 9 medium, 9 small, and 10 negligible. Source: data/results/cliff_delta_matrix.csv.

	CaSee	Deep	FiRE	RareQ	Cell	Expr	hvg	Rand	scCAD	scMal
CaSee	—	0.70	0.08	0.35	0.32	0.15	−0.83	0.68	0.41	0.21
DeepScena	−0.70	—	−0.77	−0.44	−0.46	−0.61	−0.93	−0.06	−0.43	−0.63
FiRE	−0.08	0.77	—	0.27	0.23	0.07	−0.80	0.75	0.39	0.15
RareQ	−0.35	0.44	−0.27	—	−0.04	−0.21	−0.85	0.41	0.09	−0.14
CellSIUS	−0.32	0.46	−0.23	0.04	—	−0.17	−0.85	0.43	0.13	−0.10
expr_threshold	−0.15	0.61	−0.07	0.21	0.17	—	−0.81	0.59	0.28	0.06
hvg_logreg	0.83	0.93	0.80	0.85	0.85	0.81	—	0.92	0.86	0.84
random_baseline	−0.68	0.06	−0.75	−0.41	−0.43	−0.59	−0.92	—	−0.39	−0.60
scCAD	−0.41	0.43	−0.39	−0.09	−0.13	−0.28	−0.86	0.39	—	−0.24
scMalignantFinder	−0.21	0.63	−0.15	0.14	0.10	−0.06	−0.84	0.60	0.24	—

Three findings stand out. First, the supervised ceiling hvg-logreg dominates every other method by a large effect (δ∈[0.796,0.929]) , confirming that the gap between supervised separability and unsupervised detection is practically large and not merely statistically significant. Second, FiRE’s advantage over the naive expr_threshold baseline is negligible (δ=0.068) : despite FiRE’s higher mean AP, its per-unit advantage is inconsistent, and many units show comparable performance. FiRE versus scMalignantFinder is likewise negligible (δ=0.146) , whereas FiRE versus DeepScena is large (δ=0.775) . Third, the “overlapping middle band” (expr_threshold, scMalignantFinder, CellSIUS, RareQ, and scCAD) is confirmed by small or negligible pairwise δ values within the band (e.g., RareQ vs. CellSIUS δ=0.039 and CellSIUS vs. scMalignantFinder δ=0.104 ), indicating that these methods are practically indistinguishable at the unit level despite rank differences in the median AP. The exploratory comparator CaSee shows a large effect over DeepScena (δ=0.702) and the random baseline (δ=0.677) , consistent with its higher median AP, while remaining well below the supervised ceiling (δ=−0.828) .

### 5.5 Rarity sensitivity and track-specific robustness

Rarity sensitivity has been assessed by regressing nAP against $\log_{10}\left(\pi_{u}\right)$ , where $\pi_{u}$ denotes the positive-cell prevalence in each benchmark unit (Figure 4c). Most methods show positive slopes, indicating that performance improved as rare cells became less sparse. CellSIUS shows the largest nAP slope (0.220, $p=1.78\times 10^{-20}$ ), whereas RareQ, CaSee, scMalignantFinder, scCAD, FiRE, and expr_threshold show similar positive slopes. These nAP slopes are descriptive (Johnson and Khoshgoftaar, 2019; Altalhan et al., 2025).

There is a reordering of methods in Track B versus Track A (Spearman rank correlation ρ=0.042 , p=0.907), so Track B stands as a stress test versus being incorporated into the primary leaderboard (Table 1 and Figure 5a, left side) (Cao et al., 2021). The track E hvg-logreg label noise analysis shows that the loss in mean AP is relative to a Track A mean AP of 0.9088: the hvg-logreg mean AP drops between 0.909 and 0.534 under 30% background-to-positive flips, 0.829 under 30% positive to background flips, 0.481 under 10% symmetric noise, and 0.460 under 20% symmetric noise. Conversely, the corresponding robustness indices (ranging from 0 to 1) for these analyses are 0.588, 0.912, 0.529, and 0.507 (Figure 5a, right side) (Mahalanabis et al., 2022).

**FIGURE 5:**
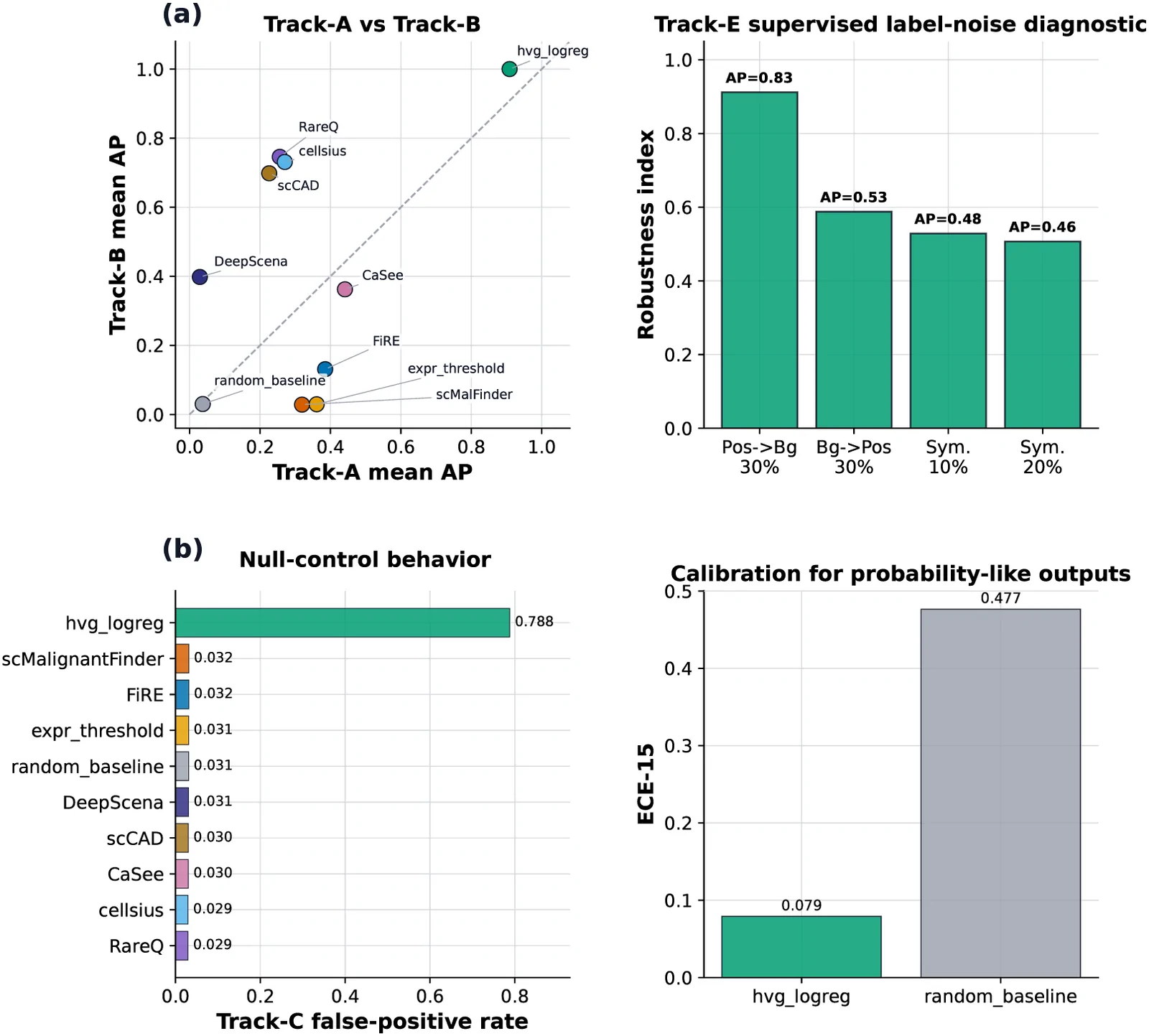
Track sensitivity, robustness, and null behavior. **(a)** Track sensitivity and supervised label noise diagnostics. Left: Track A vs. Track B mean AP per method; the dashed diagonal line (y = x) indicates equal mean AP on both tracks. Right: Track E robustness indices for the supervised ceiling under four label perturbations. Track E is not interpretable for unsupervised methods. **(b)** Track C null behavior and calibration. Background-dominated units (N=160) assess false-positive behavior; per method FPR values and pairwise statistics are reported in Table 8. All unsupervised methods converge to the 3% random floor (pairwise Wilcoxon vs. random baseline, BH-FDR >0.05 ); hvg-logreg is the sole outlier (FPR = 0.788). The Expected Calibration Error is shown only for methods with probability-like outputs.

### 5.6 Null behavior and calibration

Track C evaluates false-positive behavior in background-dominated units, where no true rare malignant cells are expected (Figure 5b; Table 8) (Torre et al., 2018). For each method, REACH measures the fraction of top-ranked cells that are incorrectly selected as positives under these null-control conditions. All eight unsupervised, exploratory, and naive scoring methods control the false-positive rate near the expected 3% floor set by the random baseline: RareQ = 0.0293, CellSIUS = 0.0295, CaSee = 0.0301, scCAD = 0.0301, DeepScena = 0.0306, random_baseline = 0.0309, expr_threshold = 0.0310, scMalignantFinder = 0.0315, and FiRE = 0.0315. The supervised hvg-logreg ceiling is the sole outlier, with a Track C false-positive rate of 0.788. The convergence of all unsupervised methods to the 3% floor on pure-null units is important for interpreting Track A: it shows that the moderate Track A AP values of outlier-based methods (e.g., FiRE 0.313 and scCAD 0.130) do not arise from indiscriminate over-flagging of background cells, but from an inability to rank malignant cells above the TME subpopulations these methods legitimately surface as outliers (see Section 7).

**TABLE 8:** Track C null-control false-positive behavior and statistical discrimination.

Method	Mean FPR	Per-unit sd	BH-FDR vs. random	Cliff’s δ
RareQ	0.0293	0.473	0.863	−0.016
CellSIUS	0.0295	0.472	0.863	−0.011
CaSee	0.0301	0.470	0.863	+0.011
scCAD	0.0301	0.472	0.863	−0.009
DeepScena	0.0306	0.471	0.964	+0.006
random_baseline	0.0309	0.471	—	—
expr_threshold	0.0310	0.468	0.863	+0.027
scMalignantFinder	0.0315	0.471	0.863	+0.004
FiRE	0.0315	0.471	0.863	+0.000
Hvg-logreg (ceiling)	0.7883	0.190	$7.0\times 10^{-27}$	−0.866

The per method mean false-positive rate (FPR) on 160 background-dominated null units, per-unit standard deviation, pairwise Wilcoxon signed-rank test against the random baseline with Benjamini–Hochberg FDR correction, and Cliff’s δ effect size. All eight unsupervised methods are statistically indistinguishable from the random baseline (BH-adjusted p>0.05 ), confirming that Track C functions as a specificity pass/fail diagnostic rather than a ranking endpoint. The supervised ceiling hvg-logreg is the sole method that differs significantly from the random baseline.

To test whether the small numerical differences among unsupervised methods carry any discriminative signal, we have performed a Friedman test on the 160 per-unit false-positive rates across all ten methods, followed by pairwise Wilcoxon signed-rank tests against the random baseline with a Benjamini–Hochberg FDR correction. The Friedman test is significant ( $\chi^{2}=545.57$ , $p<10^{-111}$ , k=10 , and N=160 ), but this significance is driven entirely by hvg-logreg: every unsupervised method fails to differ from the random baseline after FDR correction (all BH-adjusted p>0.05 , Cliff’s δ in [−0.016,0.027] ), whereas hvg-logreg differs from the random baseline with a large effect size ( $p=7.0\times 10^{-27}$ , Cliff’s δ=−0.866 ). Track C therefore does not rank unsupervised methods; it certifies that they pass the null-control floor. The high false-positive rate of hvg-logreg is expected because it is an in-sample supervised ceiling that overfits to the absence of signal on all-negative data, a known cross-validation pathology that does not affect its Track A performance. A method that cannot keep its null-control false-positive rate near the 3% floor is unfit for deployment in low-prevalence screening, and Track C is retained as a specificity pass/fail diagnostic rather than a ranking endpoint. Calibration has been assessed only for methods that produced probability-like scores (Christensen et al., 2023; Abdelaal et al., 2019).

### 5.7 Runtime, scalability, and failure modes

Runtime varies by approximately two orders of magnitude across methods (Figure 6). All runtimes have been measured on the environment described in §3.9 (AMD EPYC 7B12, 16 vCPUs, 62 GiB RAM, CPU-only), with the sole exception of DeepScena, which requires CUDA and have been run on a Google Colab Tesla T4 GPU; DeepScena’s runtime is therefore reported but is not directly comparable with the CPU-only methods. Track A mean execution times (MET) for the CPU-only tools range from 3.3 s (hvg-logistic regression) to 7.6 s (scMalignantFinder) to 357.5 s (CaSee); FiRE MET of 118.1 s, scCAD MET of 123.4 s, RareQ MET of 78.2 s, and CellSIUS MET of 39.8 s. DeepScena, measured on the Colab T4 GPU, recorded a MET of 80.6 s. Runtimes are recorded under the standardized wrapper contract, which captures wall-clock time, memory use, and per-unit resource settings in runmeta.json files (Phase 9) (Bren et al., 2022; Wang et al., 2022). Runtime also needs to be put into context with fallback and/or degenerate-output behavior. Runtime should therefore be interpreted together with fallback and degenerate-output rates because a fast method is not necessarily reliable if it frequently returns constant scores or fails on many benchmark units (Everingham et al., 2010; Tran et al., 2020).

**FIGURE 6:**
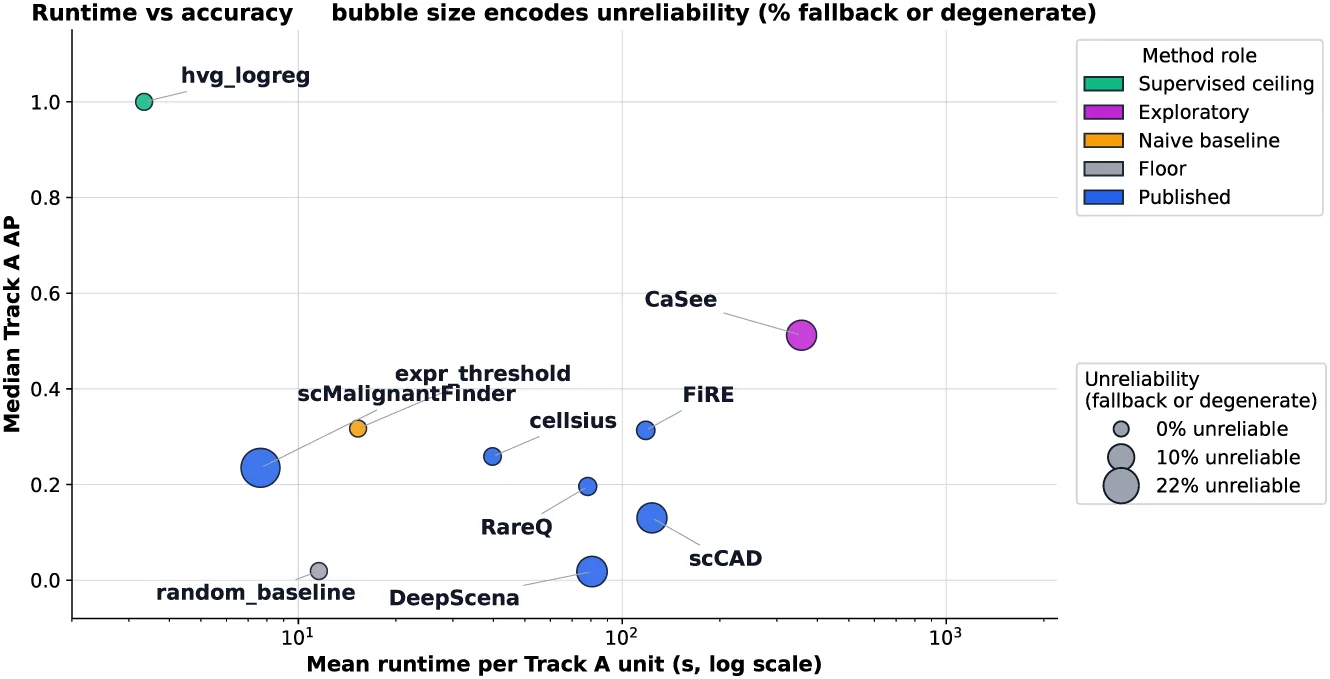
Runtime and AP Pareto behavior. Left: per-unit runtime versus cells per unit. Right: mean Track A runtime versus mean Track A AP, highlighting the trade-off between ranking performance, compute cost, and method stability. All methods were run on a CPU-only node (AMD EPYC 7B12, 16 vCPUs, 62 GiB RAM; §3.9) except for DeepScena, which has been run on a Google Colab Tesla T4 GPU and is annotated accordingly; its runtime is not directly comparable with the CPU-only methods. Runtimes are wall-clock times recorded under the standardized wrapper contract (Phase 9 runmeta.json).

## 6 Discussion

REACH shows a clear gap between supervised separability and the performance of current unsupervised rare malignant cell ranking methods (Abdelaal et al., 2019; Christensen et al., 2023). The supervised hvg-logreg ceiling indicates that many high-confidence malignant and background labels contain recoverable gene expression differences. This gap is not an artifact of conservative label construction: the ceiling collapses on ov_izar_tirosh (median AP = 0.054, where unsupervised methods outperform it) and on the intralineage Track F (median AP = 0.001, Δ=−0.999 from Track A), and it degrades under Track E label noise (mean AP 0.909 → 0.460–0.534). These three lines of evidence indicate that the ceiling tracks the interlineage malignant-versus-TME biological separability, modulated by a secondary contribution from the conservative label assignment. The lower Track A AP values obtained by unsupervised and rarity-based methods therefore reflect a real capability gap in recovering an existing biological signal, not a label design artifact (Lei et al., 2023; Märtens et al., 2023). This gap is important because rare malignant cell detection is a question of not only whether the signal exists in the data but also whether an unsupervised method can reliably recover it under strong class imbalance and tumor heterogeneity.

The results also support the need for a multitrack benchmark rather than a single leaderboard. Track A provides the primary evaluation under controlled real-cell rarity. Track B tests whether methods remain stable under synthetic Splatter stress. Its ranking does not reproduce Track A (Spearman ρ=0.042 , p=0.907 ; Table 1b)—only the supervised ceiling retains its rank (1 → 1), whereas all ranked published methods reorder substantially (e.g., RareQ 7 → 2, DeepScena 10 → 5, and scMalignantFinder 5 → 10). This near-zero correlation is the intended behavior of a stress test: it confirms that Track B probes a distinct axis of a method behavior (synthetic-data robustness) and therefore should not be averaged into the primary leaderboard. Track C adds a null-control setting that functions as a specificity pass/fail diagnostic rather than a ranking endpoint: all eight unsupervised methods converge to the 3% false-positive floor set by the random baseline and are statistically indistinguishable from it (pairwise Wilcoxon, BH-FDR >0.05 ), whereas the supervised ceiling over-flags background cells (FPR = 0.788) due to in-sample overfitting on all-negative data. A method that fails this null-control gate is unfit for low-prevalence deployment, regardless of its Track A ranking. Track D preserves natural prevalence in blood and circulating tumor cell datasets. Track E evaluates the effect of label corruption on the supervised ceiling and should not be interpreted as evidence of unsupervised robustness (Cao et al., 2021; Torre et al., 2018; Mahalanabis et al., 2022). Track F adds a further check by removing the tumor-versus-normal-tissue contrast that the other tracks rely on. Under this stricter test, nearly every method, including the supervised ceiling, drops to near-chance performance. This shows that doing well on the main task does not guarantee that a method can also spot rare cancer cells hiding within the same tissue type. The magnitude of the collapse indicates loss of practically useful discrimination, not partial degradation. At 0.1% prevalence (chance AP = 0.001), nine of 10 methods fall to median AP ≤0.009 , spanning every method class: the supervised ceiling (1.000 → 0.001), CaSee (0.512 → 0.003), FiRE (0.313 → 0.005), the naive expression baseline (0.317 → 0.009), and the remaining ranked published methods (≤0.001) . The sole exception, scMalignantFinder (median AP = 1.000), is a colorectal-cancer-specific result that cannot be generalized (Section 7). To isolate the cause, we restrict the comparison to matched crc lee units (Track A tiers T3–T4 against all Track F units), holding the positives, labeling procedure, scoring contract, metric, and unit size constant so that the background composition is the only systematic difference. The mean AUROC falls from 0.988 to 0.705 (FiRE), 0.966 to 0.789 (CaSee), 0.751 to 0.319 (CellSIUS), 0.719 to 0.365 (scCAD), and 1.000 to 0.500 (supervised ceiling). This matched contrast strongly indicates that the Track A performance of these methods depends substantially on the transcriptomic variance separating the malignant epithelial compartment from the immune and stromal lineages of the surrounding tumor microenvironment. Once that interlineage contrast is removed, no general-purpose unsupervised method in the panel retains practically useful sensitivity to rare malignant cells. The Track A leaderboard and the Tracks B–E diagnostics therefore measure interlineage rare malignant cell detection and must not be read as evidence that these tools can perform intralineage rare-cell discovery. Together, these tracks show that different evaluation conditions reveal different aspects of method behaviors.

The main practical message from REACH is that method performance should be interpreted with caution. FiRE is the strongest-ranked published comparator on the primary Track A endpoint, but its advantage is not uniform across all comparisons. In the paired tests, FiRE is not significantly better than the naive expr_threshold baseline or scMalignantFinder, and Cliff’s δ effect sizes confirm that these differences are practically negligible ( δ=0.068 vs. expr_threshold; δ=0.146 vs. scMalignantFinder), meaning that FiRE’s higher median AP does not translate to a consistent per-unit advantage. By contrast, FiRE’s advantage over DeepScena is both statistically significant and practically large (δ=0.775) , and the supervised ceiling dominates every unsupervised method by a large effect ( δ∈[0.796,0.929] ; Table 7). CaSee performs better than FiRE in the exploratory comparison (median AP 0.512 vs. 0.313 on all-units; 0.606 vs. 0.274 on the 140 common non-degenerate units, Wilcoxon $p=3.96\times 10^{-8}$ ), but it is categorized as an exploratory comparator rather than a ranked published detector because it is a cancer/normal cell scoring framework rather than a dedicated rare-cell detector, produces degenerate outputs on 12.5% of Track A units, and is approximately 3× slower than FiRE (Yuan et al., 2022; Yu et al., 2025). Dataset-level results also show that different methods perform best in different tumor contexts. Therefore, REACH does not support the idea of a single universally best method. Instead, rare malignant cell detection methods should be reported together with the dataset context, prevalence level, failure behavior, and the evaluation track in which they have been tested (Peng et al., 2019; Qian et al., 2020; Tirosh and Suva, 2024).

## 7 Limitations

This study has several limitations that should be considered when interpreting the results.

The current version of REACH uses infercnvpy (v0.6.1) as the sole copy number inference arm. To assess whether CNV evidence circularly inflates method rankings, we have rederived HC labels after dropping the CNV criterion entirely and have recomputed median AP for every method. Removing CNV collapses median AP to the chance floor (0.009–0.010) for every method, including the supervised hvg-logreg ceiling (Table 9); the ranking-level Spearman ρ between CNV-free and published rankings is 0.648 (p=0.043) , but this is computed over near-tied chance values, so only the broad rank order is preserved. This is not a circularity threat: CNV is used for labeling only, and no CNV-based detector appears on the leaderboard. To validate infercnvpy-derived CNV burden against an independent ground truth, we have compared it with published source malignancy annotations across nine datasets (57,811 cells; Table 9); CNV burden has discriminated malignant from non-malignant cells with overall AUROC = 0.782 and MCC = 0.361. CNV is therefore used as one of three independent HC label components, not as a standalone ground truth. Cross-validation with a second CNV caller (CopyKAT, Numbat, or SCEVAN) would further strengthen CNV evidence but is not feasible at this scale.

**TABLE 9:** CNV validation analyses.

Method	Median AP (CNV-free)	Median AP (published)
(a)		
hvg_logreg	0.0100	1.000
CaSee	0.0095	0.512
expr_threshold	0.0095	0.317
FiRE	0.0100	0.313
CellSIUS	0.0090	0.259
scMalignantFinder	0.0095	0.235
RareQ	0.0090	0.196
scCAD	0.0097	0.130
random_baseline	0.0090	0.019
DeepScena	0.0085	0.018
Ranking-level Spearman ρ=0.648 , p=0.043 (CNV-free vs. published ranking)		

Dataset	n_cells	n_pos	n_bg	Sens	Spec	MCC	AUROC
(b)							
bcc_yost	9,566	673	8,893	0.703	0.729	0.240	0.804
breast_ctc_szczerba	69	67	2	0.269	1.000	0.103	0.761
crc_lee	4,134	714	3,420	0.286	0.952	0.316	0.892
hcc_wei	3,228	633	2,595	0.332	0.914	0.287	0.747
hnscc_puram	1,470	323	1,147	0.749	0.970	0.763	0.906
luad_laughney	7,982	620	7,362	0.956	0.810	0.475	0.960
ov_izar_tirosh	7,141	586	6,555	0.706	0.941	0.563	0.868
pdac_peng	21,416	705	20,711	0.641	0.961	0.459	0.934
rcc_multi	2,805	689	2,116	0.261	0.933	0.262	0.759
Overall	57,811	5,010	52,801	0.556	0.895	0.361	0.782

(a) Circularity check: median Track A AP per method under CNV-bearing (published) vs. CNV-free HC labels. When the CNV criterion is dropped from label construction, every method—including the supervised hvg_logreg ceiling—collapses to the chance floor (0.009–0.010). The ranking-level Spearman correlation between the two columns is ρ=0.648 (p=0.043) , computed once across methods. Source: data/results/circularity_ranking.csv. (b) CNV concordance: infercnvpy-derived CNV burden vs. published source malignancy annotations. Per-cell mean |ΔCNV| relative to an immune/stromal reference has been used to classify cells as malignant or non-malignant and compared with the original authors’ source annotations across nine datasets (57,811 cells total). Overall AUROC =0.782 and MCC =0.361 . Source: data/results/cnv_concordance.csv.

The near-perfect Track A performance of the hvg-logreg ceiling (median AP = 1.000) reflects a mixture of biological separability and conservative label construction, and the two contributions cannot be fully separated with the current design. Three lines of evidence indicate that biological separability is the primary driver: the ceiling collapses on ov_izar_tirosh (median AP = 0.054, with unsupervised methods outperforming it), collapses on the intra-lineage Track F (median AP = 0.001, Δ=−0.999 ), and degrades under Track E label noise (mean AP 0.909 → 0.460–0.534). The ceiling also reflects massive interlineage expression variance and is trivial as a detection target: a lineage-masking scorer (EPCAM/keratin vs. immune/stromal markers) would be expected to approach this bound. The current method set lacks a pure lineage-marker baseline, so REACH cannot yet directly quantify this trivial-separation bound; the ceiling’s collapse to 0.001 on Track F confirms the interpretation (Section 5.2). Future work should add a lineage-mask baseline and a label-strictness sweep to disentangle biological separability from label conservatism.

Track E is a supervised label noise diagnostic and should not be interpreted as a general robustness test for unsupervised methods. Track C has limited discriminative power among unsupervised methods: on the 160 background-dominated null units, every method controls its false-positive rate near the 3% floor, so Track C certifies that methods do not over-flag background cells but does not rank them.

The most important limitation of the evaluated methods (not of REACH itself) is that no general-purpose unsupervised tool in the panel is demonstrably capable of intralineage malignant cell detection. Their Track A performance rests on an interlineage contrast: the transcriptomic distance from an epithelial tumor clone to the immune and stromal cells of the tumor microenvironment is large relative to any within-lineage difference. Track F removes that distance, and nine of ten methods, the supervised ceiling included, fall to near-chance average precision; the sole exception, scMalignantFinder, is a supervised pan-cancer classifier whose Track F result is colorectal-cancer-specific as discussed later. Users should therefore not deploy these tools to search for rare intralineage transcriptional states—drug-tolerant persisters, EMT or partial-EMT intermediates, transitioning or transcriptionally plastic clones, or emergent resistant subpopulations within an already-identified malignant compartment—on the basis of a Track A ranking. Track F is not itself a direct benchmark of such substates: it contrasts malignant with normal epithelium of the same broad lineage, a necessary but weaker condition than resolving substates within an already-malignant population. Track F therefore bounds these methods from above rather than measuring substate discovery directly. Detecting such states remains a distinct and currently unsolved problem; the results in Table 5 indicate that it will require methods that model the within-lineage structure explicitly rather than global rarity or anomaly.

Track F is currently restricted to a single dataset (crc_lee, colorectal cancer) because it is the only REACH dataset carrying both epithelial cell-type annotations and normal/tumor tissue origin labels; the other nine datasets lack the required normal epithelial reference. Track F therefore demonstrates the intralineage principle—that inter-lineage leaderboards cannot be extrapolated to within-lineage malignant detection—but does not yet support cross-cancer generalization. scMalignantFinder’s Track F AP = 1.000 is colorectal-cancer-specific (its Track A AP ranges from 0.015 on hnscc_puram to 0.943 on crc_lee) and must not be generalized.

The current dataset collection does not yet cover the full diversity of cancer scRNA-seq data: most datasets are based on 10× Chromium technology, only two use Smart-seq2, and spatial transcriptomics, multi-omics datasets, and a wider range of tumor types are not included. The present results should therefore be viewed as a first benchmark snapshot rather than a complete evaluation. Method stability remains an important practical issue: 249 fallback units and 377 degenerate units have been recorded, showing that some methods face difficulties related to memory use, runtime limits, wrapper stability, or constant score outputs. The assignment of methods to interpretive categories (supervised ceiling, exploratory comparator, ranked published, naive baseline, and floor baseline) is a scoping convention rather than a performance judgment, and reasonable benchmark designers could resolve edge cases differently; CaSee, for example, is categorized as an exploratory comparator yet significantly outperforms the ranked published methods on Track A.

The high-confidence label construction depends on two free parameters ( $\tau_{\text{AUCell}}$ and $\tau_{\text{kNN}}$ ); a sensitivity analysis across nine threshold combinations (Table 1a) has showed that on five of seven datasets, the P_HC label set was completely invariant to the tested thresholds, and on all datasets, no cell satisfied the full four-criteria intersection for the B_HC group $\left(n_{B_{\text{HC}}}=0\right)$ , so Track A background cells are drawn from a broader pool. REACH’s false-positive definition is task specific and may penalize biologically valid discoveries: outlier-based methods (FiRE, scCAD) will legitimately detect rare TME subpopulations that are not in the P_HC set, and these count as false positives under REACH’s malignant cell detection contract—a deliberate scope choice modulated by precision. This applies with particular force on Track F, where the cluster-decomposition methods rank background epithelial cells above the malignant spike-in (Section 5.2); their subchance Track F AUROC is consistent with a mismatch between the method’s target—cluster-level rarity—and the benchmark’s target, rather than with a failure to detect rarity as such. Finally, public leaderboards can create a risk of overfitting if methods are repeatedly tuned on the same visible datasets; future versions of REACH should include hidden test datasets, preregistered track definitions, and containerized execution with fixed prediction schemas.

## 8 Conclusion

REACH offers a reproducible way to compare methods for detecting low-abundance malignant cells against an interlineage background in scRNA-seq data. The benchmark is designed around the practical difficulties of this task: rare cells are often present at very low frequency, their labels may require more than one source of evidence, and method outputs can vary across datasets and tumor contexts. To address these issues, REACH combines multi-evidence high-confidence labels, controlled and natural prevalence settings, null controls, label noise analysis, fallback-aware metrics, statistical testing, and reproducible figure generation from audited results.

The current benchmark shows that rare malignant cell detection remains challenging. The supervised hvg-logreg ceiling performs strongly on Track A, indicating that many high-confidence malignant and background cells can be separated using gene expression information. However, current unsupervised and rarity-based methods recover only part of this separation. Among the ranked published methods, FiRE gives the strongest performance on the primary Track A endpoint, whereas CaSee provides a strong exploratory comparison (median AP 0.512, outperforming FiRE) but is categorized as an exploratory comparator rather than a ranked published detector because it is a cancer/normal cell scorer, not a dedicated rare-cell detector, and produces degenerate outputs on 12.5% of Track A units. The results also indicate that the best performing method changes from one dataset to another, suggesting that no single method is consistently best across all tumor contexts.

These findings support the need for careful, context-specific benchmarking. Instead of reducing performance to one overall leaderboard, REACH encourages results to be interpreted together with the tumor type, rarity level, false-positive behavior, method stability, and evaluation track. In this sense, REACH provides a transparent platform for future method comparison and a practical reference for developing more robust and reproducible malignant cell detection methods. Extending that reference from the inter-lineage regime characterized here to the intra-lineage regime probed by Track F—where no general-purpose unsupervised method in the current panel performs at a practically useful level—is, on the evidence of this benchmark, the more consequential open problem.

## Data Availability

The REACH benchmark framework, including all source codes, pipelines, and evaluation scripts, is publicly available at https://github.com/jaswanthmoram/reach-rarecell-benchmark. Versioned Zenodo archives containing the processed datasets, benchmark units, and result tables are available with the latest release. All datasets used in this project have been obtained from public databases, specifically GEO, and all sources are cited within the manuscript. The processed dataset, along with benchmark units and performance results, will also be available with the repository. The GitHub repository includes toy workflows, scripts for deterministic snapshot regeneration, and documentation for reproducing the benchmark using the archived data and specified compute environment. All results and figures reported in this analysis can be regenerated deterministically from any prediction output stored using the scripts provided. The full computational environment for the analyses is captured in data/results/environment/environment_capture.txt (see Section 3.9 for a summary); per method resource settings are recorded in per-unit runmeta.json files, and the original v1.2.0 preprocessing environment is documented in the repository release notes.
